# Supplementary material for: Computational Modelling Reveals Slower Safety Learning and Threat Extinction are Associated With Higher Anxiety Severity in Remote Fear Conditioning
Source: Comput Psychiatr. 2026 Jan 21;10(1):18–35. doi: 10.5334/cpsy.138 (PMC12829443; doi:10.5334/cpsy.138)

# Supplementary Methods

## Counter-factual model specification

The Rescorla-Wagner model was expanded with an additional learning rate, to model the counter-factual value updating observed from CS+ trials on to subsequent CS- trials, in acquisition phase. This updating was seen to be bi-directional, both within and across participants, i.e. the prediction error from a CS+ trial could generate either a negative or positive update on the next CS- trial, increasing or decreasing the US Expectancy Rating (ER) respectively.

Building on model 2d from the main analysis, which had two learning rates, LR^CS+^ and LR^CS-^, an additional learning rate, LR^fictive^ was applied to all CS+ trials. Unlike the other learning rates, this was in the interval [-1, 1], to allow for negative and positive value updating. This process ‘transfers’ the prediction error from CS+ to CS- trials.

$V_{t+1}^{CS+}=V_{t}^{CS+}+\left( US-V_{t}^{CS+} \right)\cdot{{LR}^{CS+}}$

$V_{t+1}^{CS-}=V_{t}^{CS-}+\left( US-V_{t}^{CS+} \right)\cdot{{LR}^{fictive}}$

To prevent VCS- exceeding the interval [0,1], it was passed through maximum and minimum functions prior to being transformed into the ordinal vector of probabilities used to estimate ER. This model is referred to as Counter-Factual Model 1.

$V^{CS-} = min\left( 1,max\left( 0,V^{CS-} \right) \right)$

To examine the possibility of the strength of counterfactual updating diminishing over trials, a further model was tested with a decay rate applied to LR^fictive^. This reduced the strength of the counterfactual update as a function of trial number. This model is referred to as Counter-Factual Model 2.

${LR}^{fictive} = {LR}^{fictive} \cdot e^{(-decay \cdot t)}$

Two further models were tested, which counterfactually updated only on surprising trials, i.e. the three unreinforced CS+ trials, without and with the decay rate applied. These models are referred to as Counter-Factual Model 3 and Counter-Factual Model 4, respectively.

Simulations and parameter recovery were performed on these four models. Results of parameter recovery are detailed in the section Supplementary Results.

# Supplementary Results

## Exclusions

Venn diagrams of exclusions indicate the numbers excluded based on one or more (intersections) criteria. For instance, many participants both reduced volume and removed headphones.


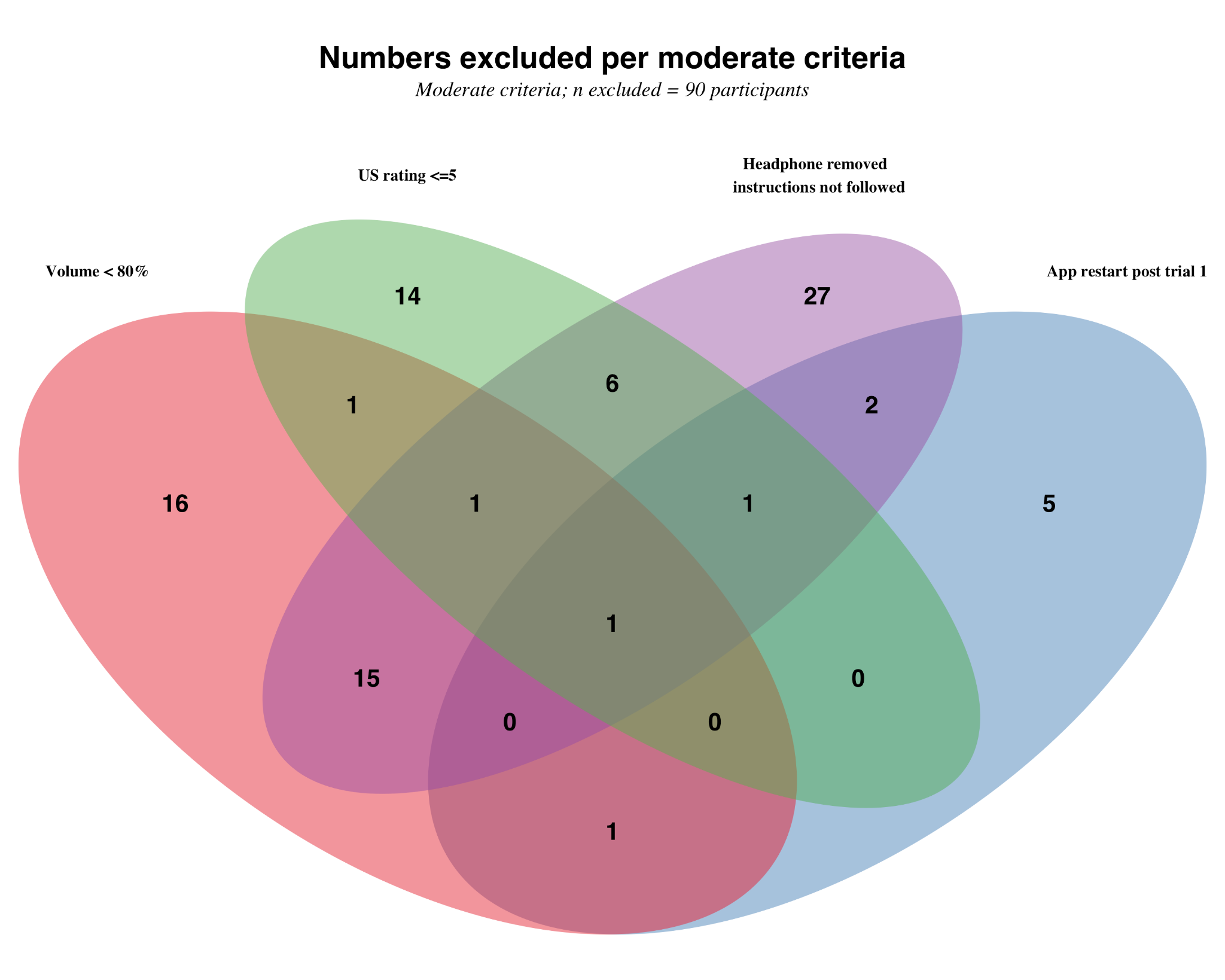


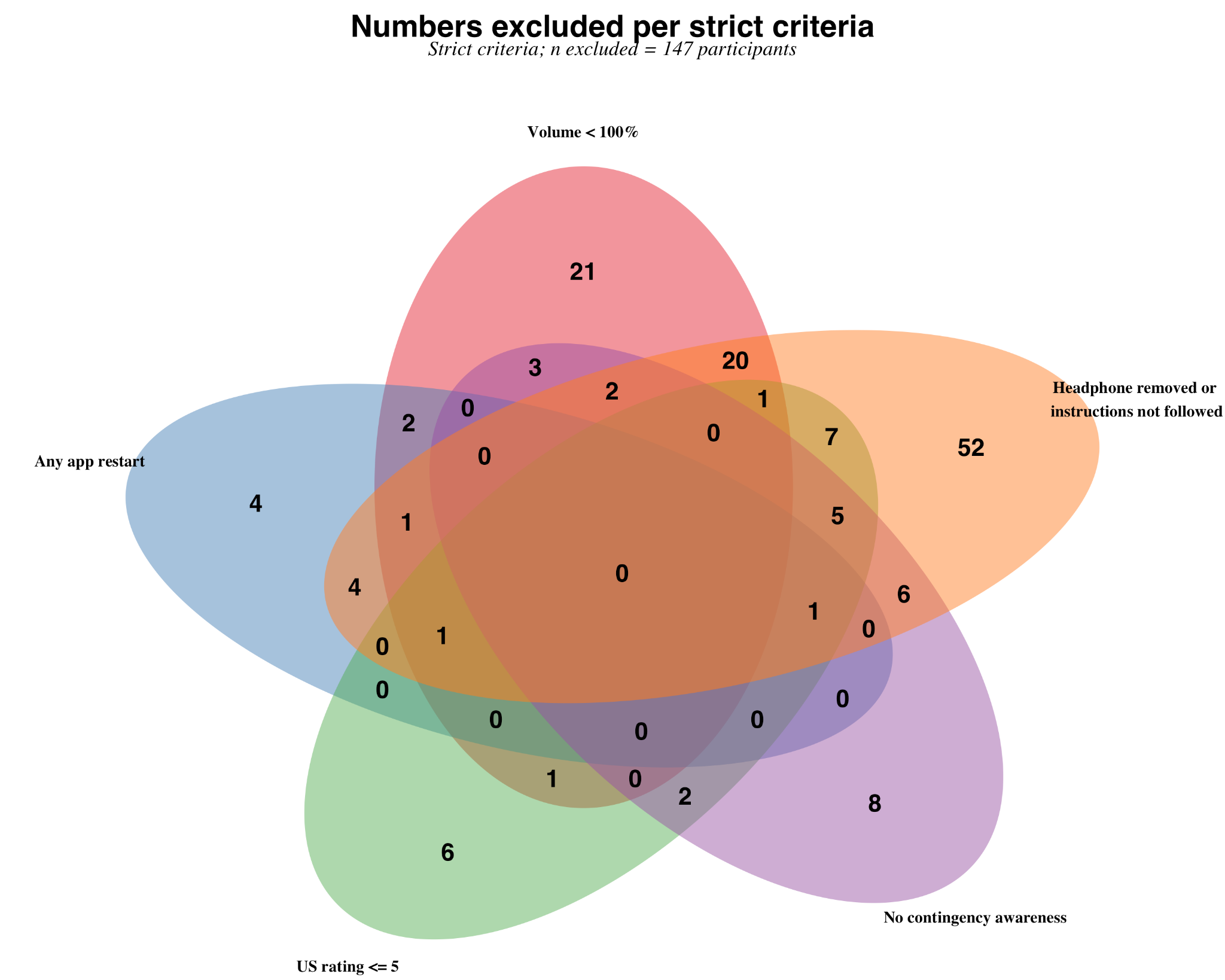


## Parameter Recovery

For each model subtype, three ranges of parameters were simulated and tested. Broad simulated parameters covering the entire parameter space (i.e. 0 to 1 for a learning rate). Low simulated parameters biased to the lower end of the space (i.e. 0 to 0.3 for a learning rate). High simulated parameters biased to the upper end of the space (i.e. 0.7 to 1 for a learning rate).

The simulated data was drawn from the CS patterns used by real participants. 145 participants were simulated each time. Recovery was assessed as successful if the known value is contained within the 95CI of the parameter estimate. Where estimates are broad and cover most of the parameter space, this metric is less useful (this is best visualised in the corresponding plots, and tends to occur for parameters based on single trials like the start parameters or CS Jump). Therefore, correlation with the known value was also calculated.

Each cell in the table shows Recovery%/Correlation. The aim was for >95% recovery for learning rate parameters.

## **Model 1**

| **Model** | **Subtype** | **Parameter_Range** | **lr** | **lapse** | **start_csp** | **start_csm** | **start_csm_jump** |
| --- | --- | --- | --- | --- | --- | --- | --- |
| 1 | a | broad | 95.2%/0.98 | 95.9%/0.89 | -/- | -/- | -/- |
| 1 | b | broad | 92.4%/0.96 | 94.5%/0.88 | 93.8%/0.67 | 98.6%/0.76 | -/- |
| 1 | c | broad | 94.5%/0.98 | 95.9%/0.88 | -/- | -/- | 95.9%/0.65 |
| 1 | d | broad | 95.9%/0.98 | 98.6%/0.89 | 91.7%/0.73 | 97.2%/0.81 | 97.2%/0.79 |
| 1 | a | high | 93.8%/0.98 | 93.8%/0.87 | -/- | -/- | -/- |
| 1 | b | high | 95.9%/0.97 | 96.6%/0.90 | 95.9%/0.51 | 99.3%/0.68 | -/- |
| 1 | c | high | 95.2%/0.97 | 96.6%/0.89 | -/- | -/- | 95.2%/0.55 |
| 1 | d | high | 96.6%/0.97 | 95.9%/0.90 | 97.9%/0.64 | 95.9%/0.70 | 96.6%/0.63 |
| 1 | a | low | 96.6%/0.98 | 95.2%/0.87 | -/- | -/- | -/- |
| 1 | b | low | 95.2%/0.99 | 95.9%/0.90 | 92.4%/0.70 | 97.9%/0.86 | -/- |
| 1 | c | low | 94.5%/0.99 | 97.9%/0.87 | -/- | -/- | 93.8%/0.79 |
| 1 | d | low | 91%/0.97 | 95.9%/0.88 | 97.9%/0.83 | 95.9%/0.88 | 93.8%/0.79 |

##

## **Model 2**

| **Model** | **Subtype** | **Parameter_Range** | **lr_csp** | **lr_csm** | **lapse** | **start_csp** | **start_csm** | **start_csm_jump** |
| --- | --- | --- | --- | --- | --- | --- | --- | --- |
| 2 | a | broad | 97.9%/0.98 | 92.4%/0.85 | 94.5%/0.87 | -/- | -/- | -/- |
| 2 | b | broad | 95.9%/0.97 | 95.2%/0.77 | 96.6%/0.89 | 95.9%/0.76 | 92.4%/0.63 | -/- |
| 2 | c | broad | 93.1%/0.97 | 97.2%/0.92 | 95.2%/0.89 | -/- | -/- | 92.4%/0.73 |
| 2 | d | broad | 94.5%/0.98 | 94.5%/0.89 | 95.9%/0.88 | 94.5%/0.69 | 95.2%/0.68 | 94.5%/0.74 |
| 2 | a | high | 95.9%/0.96 | 93.8%/0.75 | 97.9%/0.89 | -/- | -/- | -/- |
| 2 | b | high | 95.2%/0.95 | 95.2%/0.77 | 94.5%/0.85 | 94.5%/0.65 | 98.6%/0.62 | -/- |
| 2 | c | high | 96.6%/0.96 | 86.9%/0.78 | 96.6%/0.88 | -/- | -/- | 95.2%/0.69 |
| 2 | d | high | 98.6%/0.96 | 96.6%/0.77 | 93.8%/0.87 | 93.8%/0.64 | 93.8%/0.56 | 95.9%/0.69 |
| 2 | a | low | 93.8%/0.98 | 95.9%/0.93 | 94.5%/0.91 | -/- | -/- | -/- |
| 2 | b | low | 95.2%/0.98 | 93.8%/0.83 | 93.1%/0.88 | 93.8%/0.77 | 94.5%/0.60 | -/- |
| 2 | c | low | 95.2%/0.97 | 96.6%/0.96 | 95.2%/0.90 | -/- | -/- | 95.9%/0.82 |
| 2 | d | low | 96.6%/0.98 | 93.8%/0.92 | 93.1%/0.88 | 94.5%/0.75 | 95.9%/0.76 | 95.2%/0.83 |

##

## **Model 3**

| **Model** | **Subtype** | **Parameter_Range** | **lr_csp_acq** | **lr_csp_ext** | **lr_csm** | **lapse** | **start_csp** | **start_csm** | **start_csm_jump** |
| --- | --- | --- | --- | --- | --- | --- | --- | --- | --- |
| 3 | a | broad | 96.6%/0.96 | 91%/0.85 | 94.5%/0.87 | 91%/0.88 | -/- | -/- | -/- |
| 3 | b | broad | 96.6%/0.93 | 96.6%/0.90 | 95.9%/0.80 | 97.2%/0.91 | 93.8%/0.69 | 95.9%/0.71 | -/- |
| 3 | c | broad | 92.4%/0.94 | 95.2%/0.90 | 96.6%/0.88 | 96.6%/0.92 | -/- | -/- | 97.2%/0.74 |
| 3 | d | broad | 93.1%/0.92 | 93.8%/0.84 | 98.6%/0.88 | 96.6%/0.90 | 95.2%/0.74 | 97.2%/0.68 | 95.2%/0.61 |
| 3 | a | high | 93.1%/0.92 | 92.4%/0.62 | 96.6%/0.82 | 93.1%/0.88 | -/- | -/- | -/- |
| 3 | b | high | 93.1%/0.88 | 96.6%/0.77 | 96.6%/0.56 | 94.5%/0.89 | 96.6%/0.64 | 93.8%/0.51 | -/- |
| 3 | c | high | 94.5%/0.91 | 95.9%/0.76 | 93.8%/0.84 | 96.6%/0.91 | -/- | -/- | 96.6%/0.61 |
| 3 | d | high | 95.2%/0.91 | 95.2%/0.69 | 97.2%/0.80 | 95.2%/0.89 | 97.9%/0.62 | 93.8%/0.54 | 95.9%/0.72 |
| 3 | a | low | 96.6%/0.91 | 95.9%/0.95 | 95.2%/0.92 | 94.5%/0.89 | -/- | -/- | -/- |
| 3 | b | low | 96.6%/0.86 | 97.2%/0.95 | 97.2%/0.81 | 97.2%/0.89 | 96.6%/0.77 | 93.8%/0.70 | -/- |
| 3 | c | low | 97.2%/0.89 | 95.2%/0.95 | 95.2%/0.96 | 96.6%/0.92 | -/- | -/- | 93.8%/0.73 |
| 3 | d | low | 91.7%/0.86 | 96.6%/0.95 | 97.9%/0.90 | 97.2%/0.91 | 95.2%/0.73 | 95.9%/0.81 | 92.4%/0.78 |

##

## **Model 4**

| **Model** | **Subtype** | **Parameter_Range** | **lr_csp_acq** | **lr_csp_ext** | **lr_csm_acq** | **lr_csm_ext** | **lapse** | **start_csp** | **start_csm** | **start_csm_jump** |
| --- | --- | --- | --- | --- | --- | --- | --- | --- | --- | --- |
| 4 | a | broad | 95.9%/0.94 | 97.2%/0.87 | 95.2%/0.83 | 99.3%/0.03 | 97.2%/0.91 | -/- | -/- | -/- |
| 4 | b | broad | 95.9%/0.93 | 93.8%/0.86 | 94.5%/0.74 | 100%/-0.06 | 99.3%/0.90 | 96.6%/0.70 | 94.5%/0.65 | -/- |
| 4 | c | broad | 95.2%/0.96 | 94.5%/0.90 | 97.2%/0.89 | 97.2%/0.76 | 95.9%/0.91 | -/- | -/- | 96.6%/0.65 |
| 4 | d | broad | 93.1%/0.92 | 95.2%/0.88 | 94.5%/0.76 | 95.2%/0.75 | 97.2%/0.89 | 94.5%/0.68 | 95.2%/0.64 | 95.9%/0.65 |
| 4 | a | high | 96.6%/0.95 | 94.5%/0.63 | 96.6%/0.82 | 97.2%/0.12 | 97.9%/0.91 | -/- | -/- | -/- |
| 4 | b | high | 94.5%/0.89 | 91%/0.65 | 99.3%/0.69 | 99.3%/-0.06 | 97.9%/0.90 | 95.2%/0.64 | 97.9%/0.53 | -/- |
| 4 | c | high | 94.5%/0.94 | 96.6%/0.72 | 96.6%/0.82 | 96.6%/0.65 | 95.9%/0.91 | -/- | -/- | 91.7%/0.44 |
| 4 | d | high | 97.2%/0.91 | 95.2%/0.67 | 98.6%/0.70 | 93.8%/0.60 | 95.2%/0.89 | 94.5%/0.62 | 96.6%/0.55 | 95.2%/0.64 |
| 4 | a | low | 95.2%/0.95 | 96.6%/0.95 | 93.1%/0.92 | 97.2%/0.22 | 95.2%/0.90 | -/- | -/- | -/- |
| 4 | b | low | 97.9%/0.93 | 95.9%/0.94 | 95.9%/0.80 | 96.6%/0.24 | 97.9%/0.90 | 97.2%/0.75 | 96.6%/0.73 | -/- |
| 4 | c | low | 96.6%/0.91 | 95.9%/0.93 | 95.9%/0.94 | 94.5%/0.83 | 93.8%/0.90 | -/- | -/- | 95.2%/0.78 |
| 4 | d | low | 93.8%/0.90 | 97.2%/0.94 | 96.6%/0.79 | 93.8%/0.82 | 97.2%/0.90 | 95.9%/0.70 | 92.4%/0.71 | 95.2%/0.71 |

##

## **Model 5**

| **Model** | **Subtype** | **Parameter_Range** | **lr_pos** | **lr_neg** | **lapse** | **start_csp** | **start_csm** | **start_csm_jump** |
| --- | --- | --- | --- | --- | --- | --- | --- | --- |
| 5 | a | broad | 97.2%/0.96 | 94.5%/0.95 | 94.5%/0.87 | -/- | -/- | -/- |
| 5 | b | broad | 97.2%/0.96 | 93.1%/0.94 | 96.6%/0.89 | 96.6%/0.66 | 92.4%/0.70 | -/- |
| 5 | c | broad | 94.5%/0.96 | 96.6%/0.96 | 95.9%/0.90 | -/- | -/- | 93.1%/0.76 |
| 5 | d | broad | 95.2%/0.95 | 95.9%/0.97 | 95.2%/0.89 | 92.4%/0.63 | 93.8%/0.74 | 93.8%/0.81 |
| 5 | a | high | 96.6%/0.93 | 95.2%/0.92 | 95.2%/0.88 | -/- | -/- | -/- |
| 5 | b | high | 96.6%/0.93 | 96.6%/0.92 | 92.4%/0.84 | 92.4%/0.65 | 95.2%/0.63 | -/- |
| 5 | c | high | 99.3%/0.94 | 94.5%/0.95 | 95.9%/0.88 | -/- | -/- | 95.2%/0.70 |
| 5 | d | high | 97.9%/0.93 | 96.6%/0.94 | 94.5%/0.84 | 93.1%/0.65 | 95.9%/0.60 | 97.2%/0.76 |
| 5 | a | low | 94.5%/0.96 | 92.4%/0.98 | 94.5%/0.90 | -/- | -/- | -/- |
| 5 | b | low | 93.1%/0.93 | 95.9%/0.97 | 93.1%/0.86 | 93.1%/0.67 | 93.1%/0.76 | -/- |
| 5 | c | low | 94.5%/0.94 | 93.8%/0.98 | 95.2%/0.90 | -/- | -/- | 94.5%/0.84 |
| 5 | d | low | 92.4%/0.92 | 95.9%/0.97 | 95.2%/0.88 | 93.8%/0.68 | 94.5%/0.81 | 94.5%/0.89 |

##

## **Model 6**

| **Model** | **Subtype** | **Parameter_Range** | **lr_csp_pos** | **lr_csp_neg** | **lr_csm** | **lapse** | **start_csp** | **start_csm** | **start_csm_jump** |
| --- | --- | --- | --- | --- | --- | --- | --- | --- | --- |
| 6 | a | broad | 95.9%/0.95 | 93.8%/0.91 | 93.8%/0.87 | 93.1%/0.87 | -/- | -/- | -/- |
| 6 | b | broad | 93.8%/0.93 | 96.6%/0.94 | 95.9%/0.80 | 98.6%/0.92 | 93.1%/0.68 | 97.2%/0.70 | -/- |
| 6 | c | broad | 94.5%/0.96 | 97.9%/0.95 | 97.2%/0.88 | 96.6%/0.93 | -/- | -/- | 97.2%/0.74 |
| 6 | d | broad | 95.2%/0.95 | 92.4%/0.92 | 97.9%/0.88 | 95.9%/0.90 | 96.6%/0.73 | 97.9%/0.68 | 95.9%/0.62 |
| 6 | a | high | 95.2%/0.85 | 97.2%/0.90 | 97.2%/0.83 | 93.8%/0.90 | -/- | -/- | -/- |
| 6 | b | high | 95.2%/0.88 | 94.5%/0.89 | 95.9%/0.56 | 93.1%/0.89 | 95.9%/0.60 | 95.2%/0.51 | -/- |
| 6 | c | high | 95.2%/0.91 | 95.9%/0.92 | 95.2%/0.84 | 95.9%/0.91 | -/- | -/- | 96.6%/0.60 |
| 6 | d | high | 94.5%/0.92 | 95.2%/0.87 | 97.9%/0.80 | 96.6%/0.90 | 97.2%/0.58 | 93.8%/0.55 | 96.6%/0.72 |
| 6 | a | low | 95.2%/0.94 | 93.8%/0.93 | 95.9%/0.92 | 95.2%/0.89 | -/- | -/- | -/- |
| 6 | b | low | 95.2%/0.90 | 94.5%/0.94 | 96.6%/0.81 | 95.2%/0.89 | 97.9%/0.73 | 95.2%/0.71 | -/- |
| 6 | c | low | 95.2%/0.93 | 93.8%/0.96 | 95.9%/0.96 | 97.2%/0.92 | -/- | -/- | 93.1%/0.73 |
| 6 | d | low | 94.5%/0.90 | 95.9%/0.95 | 95.9%/0.90 | 96.6%/0.91 | 97.9%/0.67 | 97.2%/0.81 | 93.8%/0.78 |

##

## **Model 7**

| **Model** | **Subtype** | **Parameter_Range** | **lr_csp_acq_us1** | **lr_csp_acq_us0** | **lr_csp_ext** | **lr_csm_acq** | **lr_csm_ext** | **lapse** | **start_csp** | **start_csm** | **start_csm_jump** |
| --- | --- | --- | --- | --- | --- | --- | --- | --- | --- | --- | --- |
| 7 | a | broad | 97.2%/0.94 | 96.6%/0.84 | 96.6%/0.87 | 93.1%/0.84 | 100%/0.19 | 97.9%/0.91 | -/- | -/- | -/- |
| 7 | b | broad | 94.5%/0.94 | 96.6%/0.84 | 98.6%/0.85 | 97.9%/0.81 | 100%/0.03 | 95.9%/0.89 | 92.4%/0.56 | 96.6%/0.65 | -/- |
| 7 | c | broad | 96.6%/0.95 | 97.9%/0.72 | 97.2%/0.86 | 97.9%/0.91 | 92.4%/0.77 | 97.9%/0.90 | -/- | -/- | 91.7%/0.58 |
| 7 | d | broad | 95.9%/0.94 | 97.2%/0.76 | 98.6%/0.85 | 99.3%/0.80 | 96.6%/0.84 | 97.2%/0.90 | 94.5%/0.56 | 97.9%/0.71 | 93.1%/0.58 |
| 7 | a | high | 95.9%/0.94 | 93.1%/0.76 | 95.9%/0.69 | 95.2%/0.83 | 98.6%/-0.11 | 95.2%/0.92 | -/- | -/- | -/- |
| 7 | b | high | 94.5%/0.88 | 97.2%/0.75 | 95.2%/0.63 | 91.7%/0.60 | 97.9%/-0.16 | 98.6%/0.90 | 96.6%/0.64 | 95.9%/0.46 | -/- |
| 7 | c | high | 95.9%/0.93 | 93.1%/0.72 | 95.9%/0.77 | 96.6%/0.75 | 95.2%/0.65 | 95.9%/0.88 | -/- | -/- | 97.9%/0.56 |
| 7 | d | high | 95.2%/0.92 | 95.2%/0.77 | 93.1%/0.71 | 93.8%/0.67 | 93.8%/0.70 | 98.6%/0.90 | 93.1%/0.57 | 96.6%/0.72 | 92.4%/0.63 |
| 7 | a | low | 94.5%/0.92 | 96.6%/0.68 | 95.9%/0.93 | 91%/0.93 | 97.9%/0.27 | 95.9%/0.89 | -/- | -/- | -/- |
| 7 | b | low | 93.1%/0.91 | 97.9%/0.74 | 97.2%/0.94 | 92.4%/0.79 | 100%/0.27 | 94.5%/0.90 | 95.2%/0.66 | 95.2%/0.64 | -/- |
| 7 | c | low | 97.2%/0.93 | 95.2%/0.63 | 93.1%/0.93 | 93.1%/0.92 | 95.9%/0.84 | 93.8%/0.88 | -/- | -/- | 97.2%/0.68 |
| 7 | d | low | 95.9%/0.90 | 92.4%/0.66 | 93.8%/0.93 | 97.2%/0.83 | 92.4%/0.86 | 96.6%/0.89 | 95.2%/0.73 | 98.6%/0.74 | 95.9%/0.74 |

##

## **Counter-factual model 1 (fictive update on all CS+ trials)**

| **param_range** | **lr_csm** | **lr_csp** | **lr_fic** | **decay_rate** | **lapse** | **start_csp** | **start_csm** | **start_csm_jump** |
| --- | --- | --- | --- | --- | --- | --- | --- | --- |
| broad | 95.9%/0.97 | 93.8%/1.00 | 96.6%/0.96 | -/- | 100%/0.31 | 95.2%/0.98 | 93.8%/0.94 | 95.2%/0.96 |
| high | 93.8%/0.98 | 91.7%/0.99 | 93.1%/0.99 | -/- | 98.6%/0.30 | 95.2%/0.98 | 91.7%/0.93 | 96.6%/0.96 |
| low | 94.5%/0.93 | 91.7%/1.00 | 95.9%/0.75 | -/- | 99.3%/0.42 | 95.9%/0.98 | 97.2%/0.94 | 97.2%/0.98 |

## **Counter-factual model 2 (fictive update on all CS+ trials & decay on fictive learning rate)**

| **param_range** | **lr_csm** | **lr_csp** | **lr_fic** | **decay_rate** | **lapse** | **start_csp** | **start_csm** | **start_csm_jump** |
| --- | --- | --- | --- | --- | --- | --- | --- | --- |
| broad | 95.2%/0.96 | 94.5%/1.00 | 98.6%/0.62 | 97.9%/0.47 | 98.6%/0.19 | 94.5%/0.98 | 97.2%/0.94 | 93.8%/0.97 |
| high | 96.6%/0.96 | 93.1%/0.99 | 99.3%/0.49 | 98.6%/0.42 | 100%/0.29 | 95.9%/0.98 | 95.9%/0.96 | 95.2%/0.98 |
| low | 92.4%/0.90 | 97.2%/1.00 | 100%/0.50 | 97.2%/0.23 | 98.6%/0.59 | 93.1%/0.98 | 93.1%/0.93 | 96.6%/0.97 |

## **Counter-factual model 3 (fictive update only on CS+US- trials)**

| **param_range** | **lr_csm** | **lr_csp** | **lr_fic** | **decay_rate** | **lapse** | **start_csp** | **start_csm** | **start_csm_jump** |
| --- | --- | --- | --- | --- | --- | --- | --- | --- |
| broad | 96.6%/0.98 | 94.5%/1.00 | 94.5%/0.94 | -/- | 97.9%/0.24 | 94.5%/0.98 | 98.6%/0.98 | 97.9%/0.95 |
| high | 94.5%/0.99 | 95.2%/0.99 | 92.4%/0.98 | -/- | 99.3%/0.41 | 89.7%/0.97 | 95.2%/0.99 | 92.4%/0.87 |
| low | 95.2%/0.92 | 91%/1.00 | 97.9%/0.67 | -/- | 99.3%/0.43 | 93.8%/0.99 | 94.5%/0.95 | 95.9%/0.96 |

## **Counter-factual model 4 (fictive update only on CS+US- trials & decay on fictive learning rate)**

| **param_range** | **lr_csm** | **lr_csp** | **lr_fic** | **decay_rate** | **lapse** | **start_csp** | **start_csm** | **start_csm_jump** |
| --- | --- | --- | --- | --- | --- | --- | --- | --- |
| broad | 95.2%/0.97 | 93.8%/1.00 | 100%/0.53 | 97.9%/0.43 | 98.6%/0.20 | 95.2%/0.98 | 98.6%/0.98 | 94.5%/0.96 |
| high | 96.6%/0.98 | 93.8%/0.99 | 95.9%/0.25 | 95.2%/0.41 | 100%/0.35 | 95.9%/0.98 | 94.5%/0.98 | 95.9%/0.98 |
| low | 94.5%/0.87 | 98.6%/1.00 | 100%/0.48 | 97.9%/0.21 | 99.3%/0.58 | 93.8%/0.98 | 93.8%/0.95 | 97.2%/0.97 |

## Prior Predictive Checks

Prior predictive check for model with single learning rate and no extra fitting parameters (model 1a). This fails to provide model space for participant behaviour in some sections


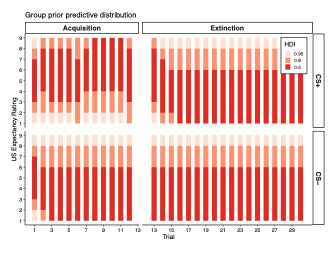


Prior predictive check for the winning model, with five learning rate parameters and three fitting parameters (model 7d). This provides model space for participant data to be modelled accurately.


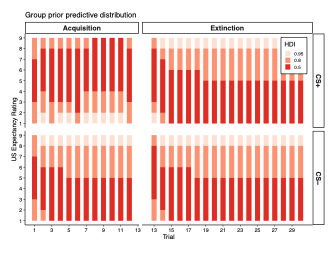


## Model Comparison

Bar chart indicating LOOIC for each model. Black lines represent the best fitting in each model class (between variants a, b, c, and d). 7d was the best fitting model across all models, with lowest LOOIC.


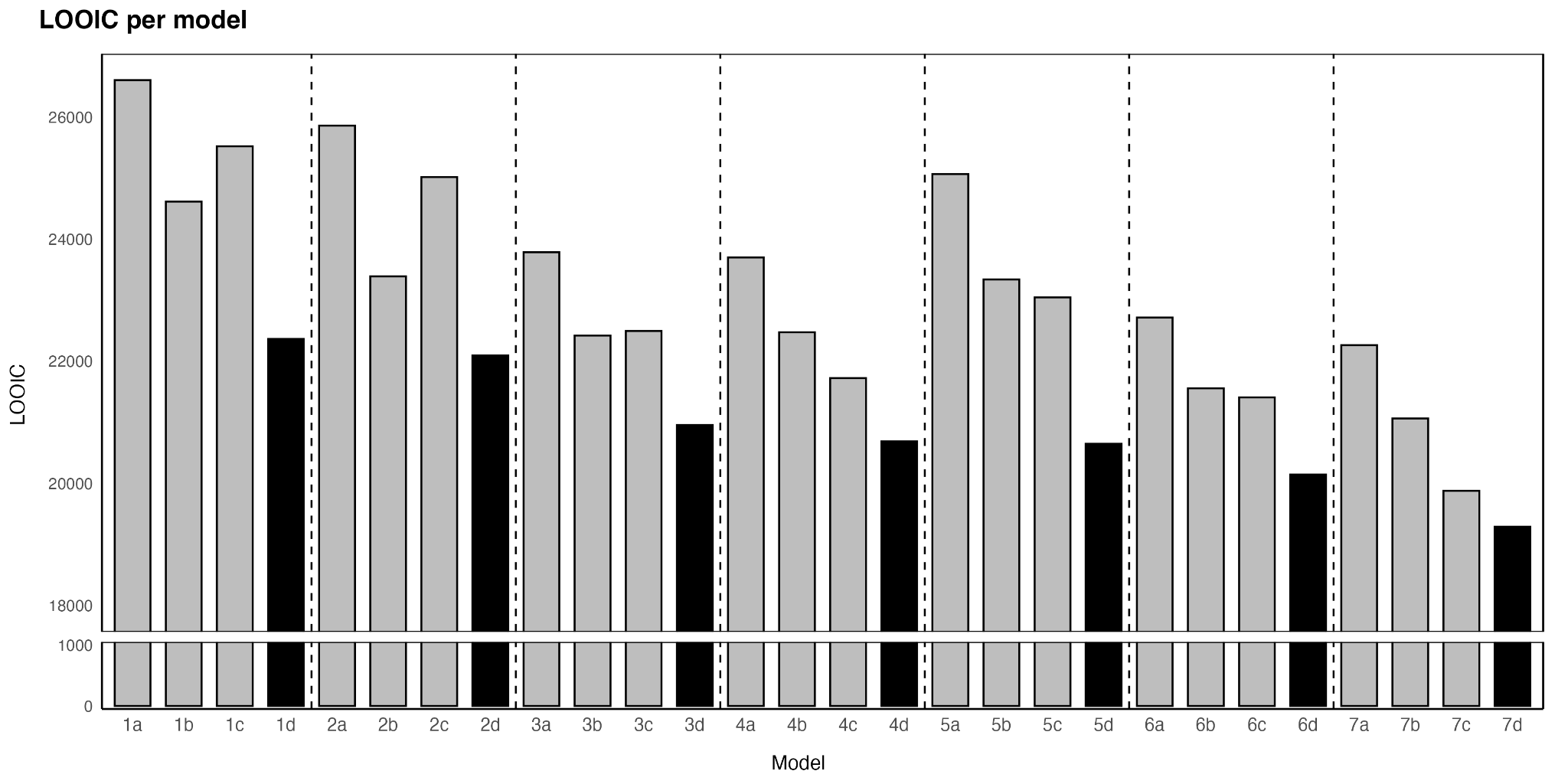


Overall model comparison table. Bold indicates the best metric i.e. highest likelihood or pseudo r2, or lowest LOOIC.
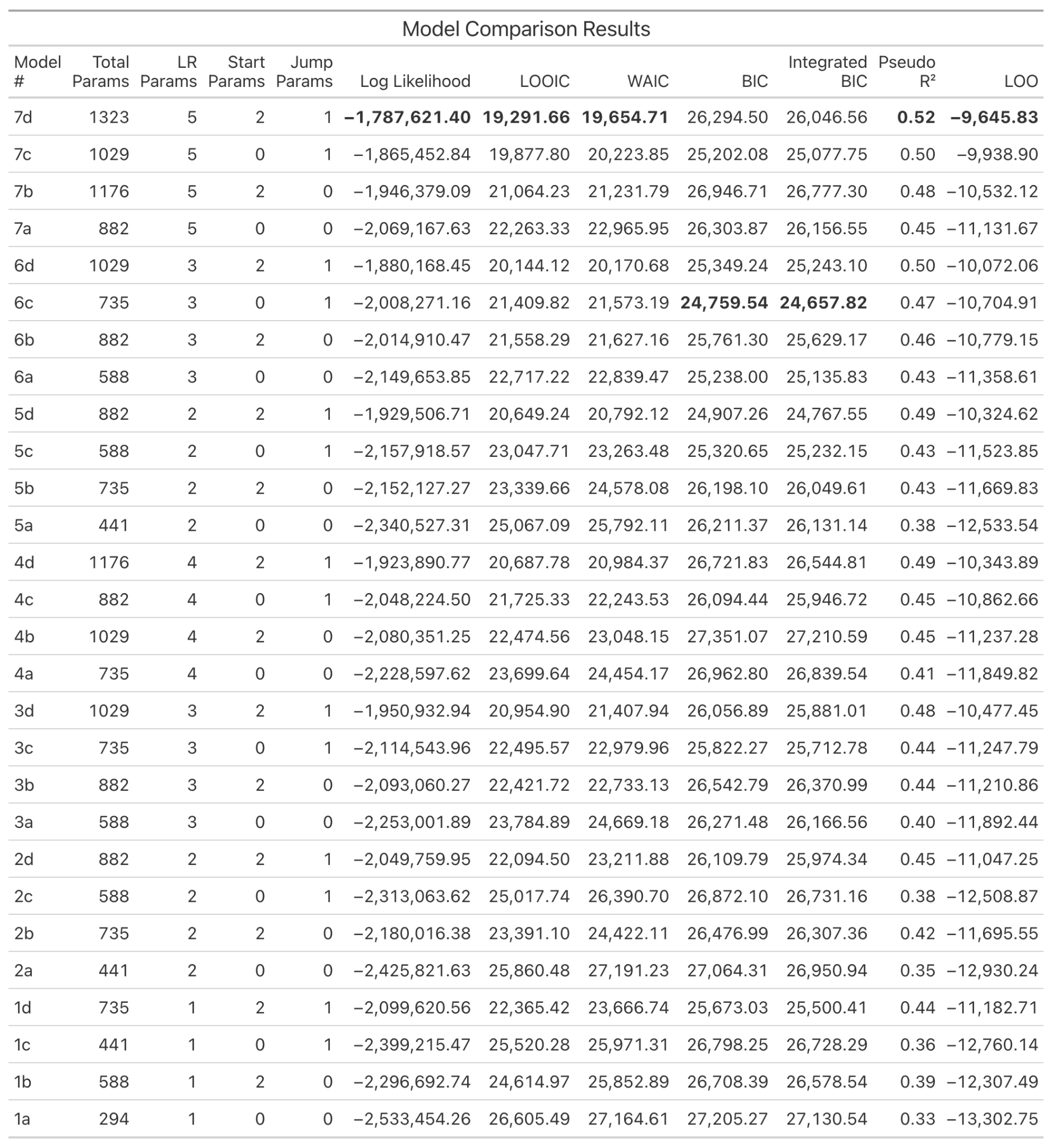


Model comparison table for acquisition CS+ trials.
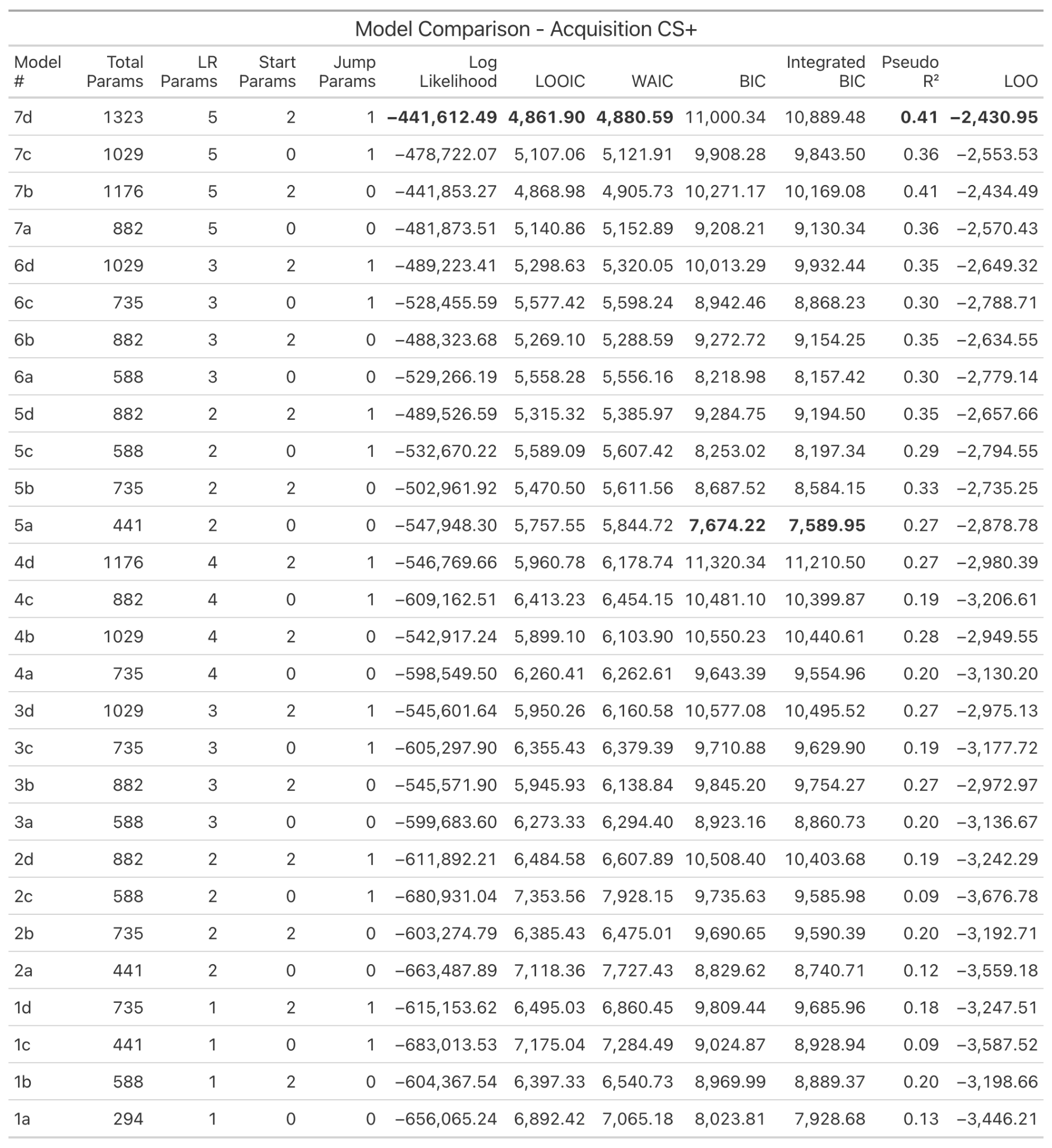


Model comparison table for acquisition CS- trials.
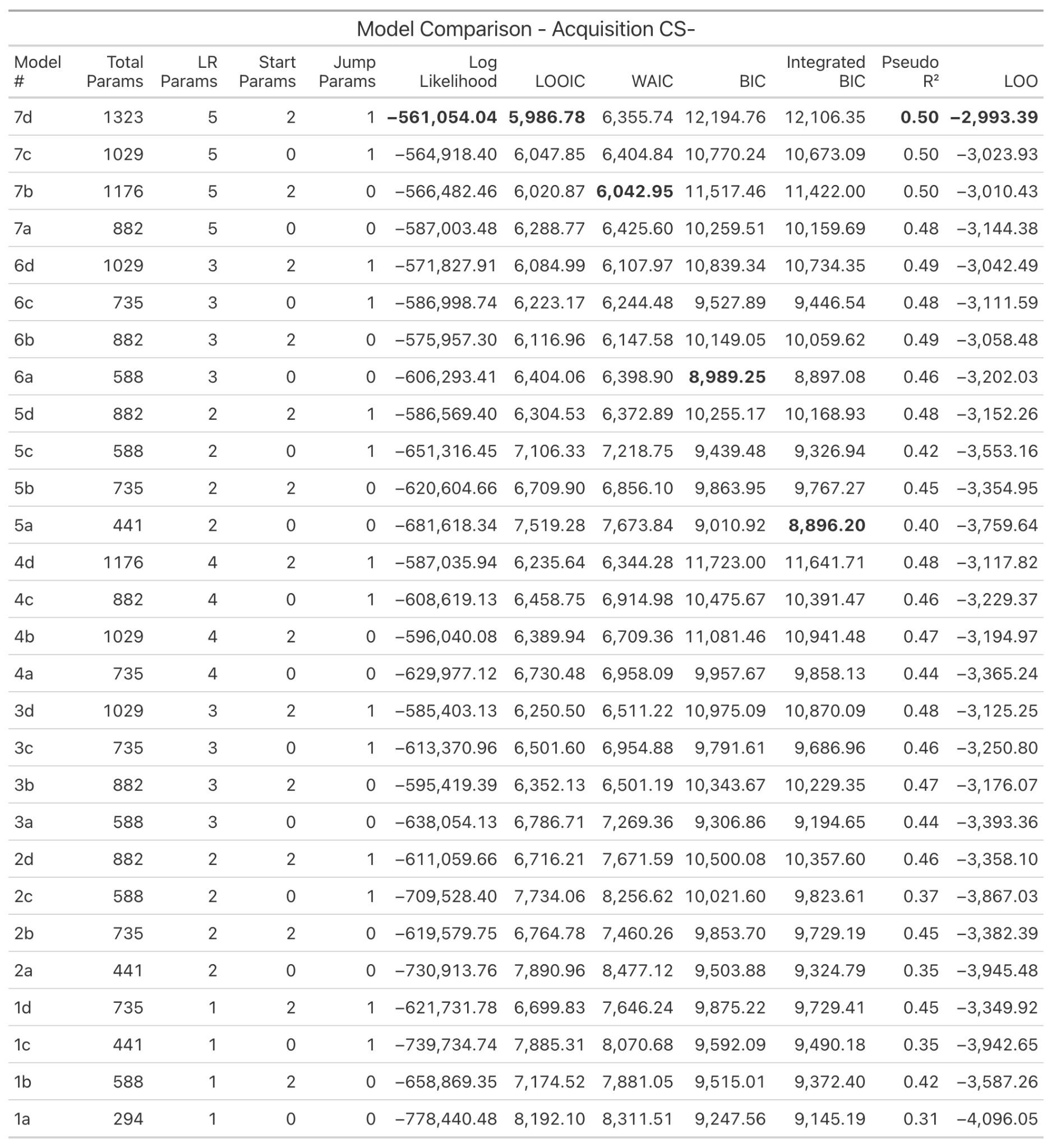


Model comparison table for Extinction CS+ trials.
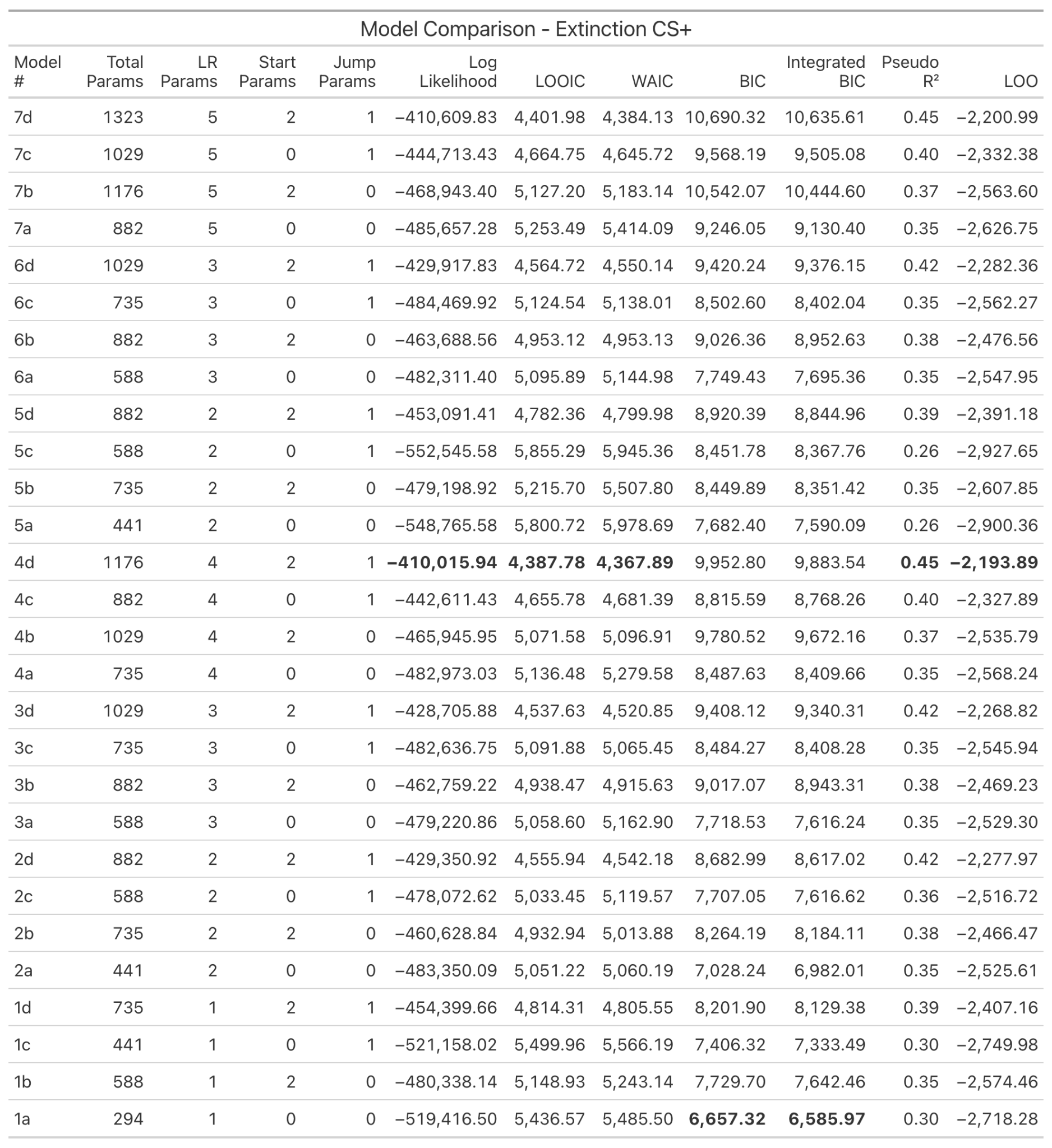
Model comparison table for Extinction CS- trials.
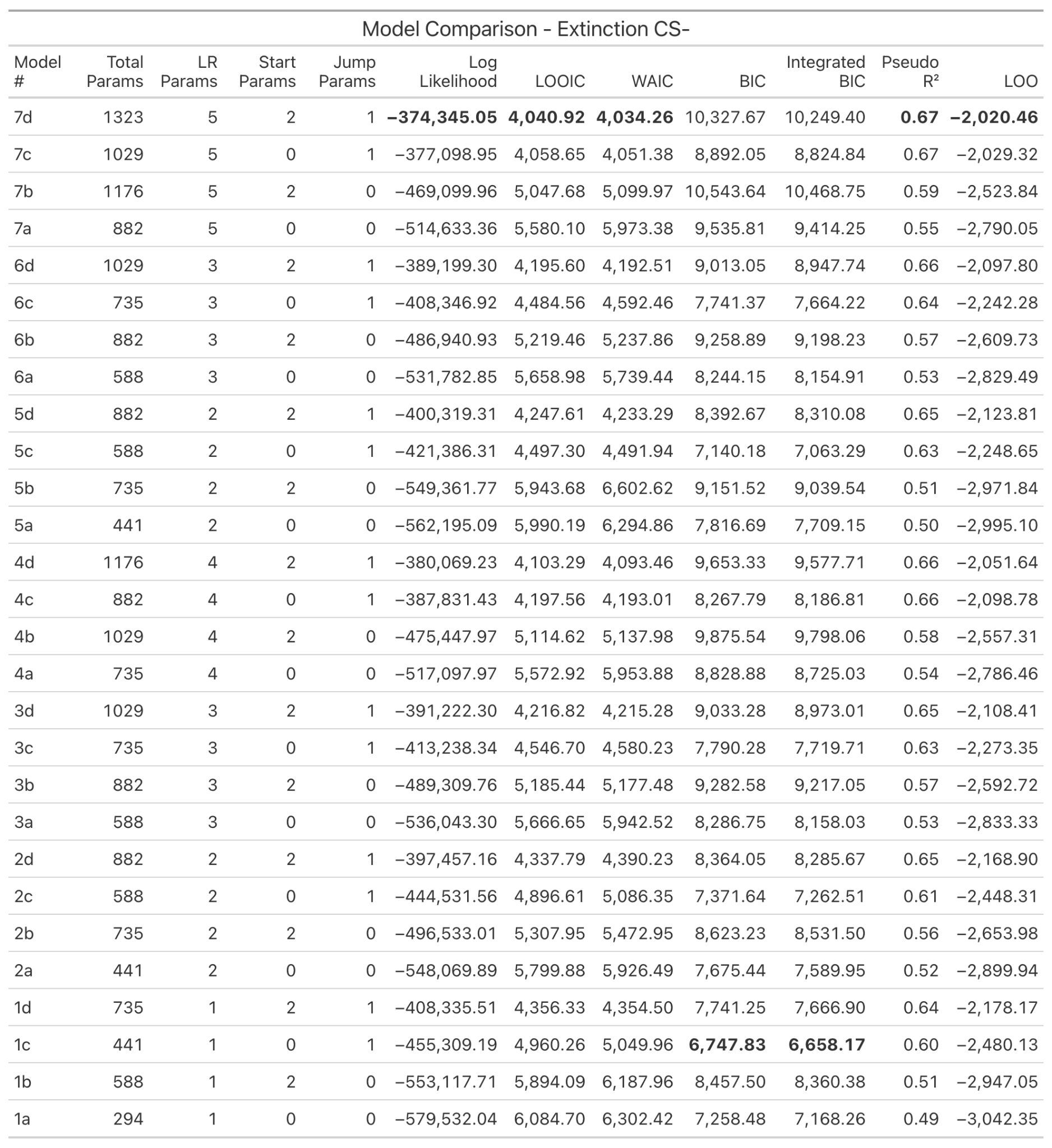


## Associations with corrections

Partial Spearman correlation, correcting for CS pattern. (Was one of the four pseudorandom CS patterns in some way influential on the association?)

|  | Medium GAD-7 | | Medium PHQ-8 | | Strict GAD-7 | | Strict PHQ-8 | |
| --- | --- | --- | --- | --- | --- | --- | --- | --- |
| Parameter | Regular | CS Pattern | Regular | CS Pattern | Regular | CS Pattern | Regular | CS Pattern |
| Acquisition CS+ Learning Rate (US+) | -0.07 [-0.23, 0.10] | -0.09 [-0.25, 0.08] | -0.03 [-0.20, 0.13] | -0.04 [-0.21, 0.12] | -0.15 [-0.36, 0.08] | -0.16 [-0.36, 0.06] | -0.01 [-0.22, 0.20] | -0.01 [-0.23, 0.20] |
| Acquisition CS+ Learning Rate (US-) | -0.10 [-0.27, 0.07] | -0.11 [-0.27, 0.07] | -0.06 [-0.23, 0.12] | -0.06 [-0.22, 0.10] | **-0.30 [-0.50, -0.07]** | **-0.30 [-0.50, -0.09]** | -0.20 [-0.40, 0.00] | -0.20 [-0.41, 0.02] |
| Acquisition CS- Learning Rate | **-0.22 [-0.36, -0.06]** | **-0.22 [-0.37, -0.06]** | -0.14 [-0.29, 0.02] | -0.14 [-0.28, 0.02] | **-0.32 [-0.49, -0.12]** | **-0.32 [-0.50, -0.11]** | -0.14 [-0.33, 0.05] | -0.14 [-0.33, 0.06] |
| Extinction CS+ Learning Rate | **-0.21 [-0.37, -0.06]** | **-0.23 [-0.38, -0.06]** | **-0.23 [-0.38, -0.06]** | **-0.23 [-0.39, -0.06]** | **-0.33 [-0.52, -0.11]** | **-0.34 [-0.53, -0.13]** | **-0.26 [-0.46, -0.06]** | **-0.26 [-0.45, -0.05]** |
| Extinction CS- Learning Rate | -0.07 [-0.22, 0.09] | -0.07 [-0.23, 0.09] | -0.10 [-0.27, 0.07] | -0.10 [-0.26, 0.07] | -0.15 [-0.35, 0.08] | -0.15 [-0.36, 0.07] | -0.13 [-0.34, 0.10] | -0.13 [-0.34, 0.10] |
| Lapse Rate | -0.06 [-0.21, 0.11] | -0.06 [-0.21, 0.10] | -0.16 [-0.31, 0.01] | -0.16 [-0.30, 0.02] | -0.13 [-0.33, 0.09] | -0.13 [-0.33, 0.09] | **-0.22 [-0.42, -0.00]** | **-0.22 [-0.42, -0.00]** |
| ***Bold ~ p < 0.05*** | | | | | | | | |
| *Regular = no controls; CS Pattern = controlling for CS pattern* | | | | | | | | |
| *GAD-7 - Generalized Anxiety Disorder seven item scale; PHQ-8 - Patient Health Questionnaire eight item scale* | | | | | | | | |
| *CS pattern distribution: 1 = 31, 2 = 40, 3 = 37, 4 = 37* | | | | | | | | |
| *Medium dataset: CS pattern correlations GAD-7 ρ=-0.08, PHQ-8 ρ=-0.02* | | | | | | | | |
| *Strict dataset: CS pattern correlations GAD-7 ρ=0.00, PHQ-8 ρ=-0.03* | | | | | | | | |

Partial Spearman correlation, correcting for age, sex, and both combined.

|  | Medium GAD-7 | | | | Medium PHQ-8 | | | | Strict GAD-7 | | | | Strict PHQ-8 | | | |
| --- | --- | --- | --- | --- | --- | --- | --- | --- | --- | --- | --- | --- | --- | --- | --- | --- |
| Parameter | Regular | Age | Sex | Both | Regular | Age | Sex | Both | Regular | Age | Sex | Both | Regular | Age | Sex | Both |
| Acquisition CS+ Learning Rate (US+) | -0.07 [-0.24, 0.10] | -0.07 [-0.23, 0.11] | -0.07 [-0.24, 0.08] | -0.07 [-0.24, 0.09] | -0.03 [-0.19, 0.13] | -0.04 [-0.21, 0.12] | -0.04 [-0.21, 0.13] | -0.04 [-0.21, 0.12] | -0.15 [-0.36, 0.05] | -0.15 [-0.37, 0.07] | -0.16 [-0.37, 0.06] | -0.16 [-0.37, 0.07] | -0.01 [-0.22, 0.21] | -0.01 [-0.23, 0.21] | -0.01 [-0.22, 0.20] | -0.01 [-0.24, 0.22] |
| Acquisition CS+ Learning Rate (US-) | -0.10 [-0.26, 0.07] | -0.08 [-0.26, 0.08] | -0.08 [-0.25, 0.09] | -0.07 [-0.24, 0.11] | -0.06 [-0.22, 0.12] | -0.03 [-0.19, 0.15] | -0.05 [-0.22, 0.12] | -0.02 [-0.20, 0.15] | **-0.30 [-0.50, -0.07]** | **-0.28 [-0.50, -0.05]** | **-0.29 [-0.47, -0.06]** | **-0.27 [-0.47, -0.06]** | -0.20 [-0.40, 0.00] | -0.17 [-0.37, 0.05] | -0.18 [-0.41, 0.04] | -0.16 [-0.37, 0.07] |
| Acquisition CS- Learning Rate | **-0.22 [-0.37, -0.05]** | **-0.22 [-0.37, -0.05]** | **-0.22 [-0.37, -0.05]** | **-0.22 [-0.36, -0.05]** | -0.14 [-0.29, 0.03] | -0.13 [-0.30, 0.03] | -0.14 [-0.30, 0.03] | -0.13 [-0.29, 0.03] | **-0.32 [-0.49, -0.12]** | **-0.31 [-0.49, -0.10]** | **-0.32 [-0.49, -0.12]** | **-0.31 [-0.50, -0.10]** | -0.14 [-0.33, 0.05] | -0.10 [-0.30, 0.11] | -0.15 [-0.34, 0.07] | -0.11 [-0.32, 0.12] |
| Extinction CS+ Learning Rate | **-0.21 [-0.36, -0.05]** | **-0.21 [-0.36, -0.04]** | **-0.21 [-0.37, -0.05]** | **-0.21 [-0.37, -0.04]** | **-0.23 [-0.38, -0.07]** | **-0.23 [-0.39, -0.05]** | **-0.22 [-0.38, -0.05]** | **-0.23 [-0.38, -0.06]** | **-0.33 [-0.52, -0.14]** | **-0.33 [-0.52, -0.11]** | **-0.33 [-0.52, -0.11]** | **-0.32 [-0.52, -0.12]** | **-0.26 [-0.44, -0.05]** | **-0.25 [-0.45, -0.03]** | **-0.25 [-0.43, -0.05]** | **-0.24 [-0.43, -0.04]** |
| Extinction CS- Learning Rate | -0.07 [-0.23, 0.10] | -0.09 [-0.25, 0.06] | -0.06 [-0.22, 0.11] | -0.08 [-0.25, 0.08] | -0.10 [-0.25, 0.07] | -0.12 [-0.28, 0.06] | -0.09 [-0.25, 0.08] | -0.12 [-0.28, 0.04] | -0.15 [-0.35, 0.07] | -0.19 [-0.39, 0.05] | -0.14 [-0.34, 0.10] | -0.17 [-0.38, 0.06] | -0.13 [-0.36, 0.09] | -0.17 [-0.38, 0.06] | -0.12 [-0.34, 0.10] | -0.15 [-0.36, 0.07] |
| Lapse Rate | -0.06 [-0.21, 0.12] | -0.06 [-0.23, 0.11] | -0.05 [-0.23, 0.10] | -0.06 [-0.23, 0.11] | -0.16 [-0.32, 0.00] | -0.15 [-0.32, 0.02] | -0.15 [-0.30, 0.01] | -0.15 [-0.29, 0.02] | -0.13 [-0.33, 0.09] | -0.14 [-0.33, 0.10] | -0.13 [-0.32, 0.10] | -0.13 [-0.35, 0.09] | **-0.22 [-0.41, -0.01]** | -0.21 [-0.42, 0.01] | **-0.21 [-0.41, 0.01]** | -0.21 [-0.40, -0.00] |
| ***Bold ~ p < 0.05*** | | | | | | | | | | | | | | | | |
| *Regular = no controls; Age = controlling for age; Sex = controlling for sex; Both = controlling for age and sex* | | | | | | | | | | | | | | | | |
| *GAD-7 - Generalized Anxiety Disorder seven item scale; PHQ-8 - Patient Health Questionnaire eight item scale* | | | | | | | | | | | | | | | | |
| *Medium dataset (n=145): Age correlations GAD-7 ρ=-0.01, PHQ-8 ρ=-0.09; Sex correlations GAD-7 ρ=0.11, PHQ-8 ρ=0.06* | | | | | | | | | | | | | | | | |
| *Strict dataset (n=88): Age correlations GAD-7 ρ=-0.06, PHQ-8 ρ=-0.21; Sex correlations GAD-7 ρ=0.09, PHQ-8 ρ=0.11* | | | | | | | | | | | | | | | | |

Partial Spearman correlation, correcting for US unpleasantness rating in medium excluded dataset (n=145)

|  | GAD-7 | | PHQ-8 | |
| --- | --- | --- | --- | --- |
| Parameter | Regular ρ [95% CI] | Partial ρ [95% CI] | Regular ρ [95% CI] | Partial ρ [95% CI] |
| Acquisition CS+ Learning Rate (US+) | -0.07 [-0.24, 0.11] | -0.09 [-0.25, 0.08] | -0.03 [-0.20, 0.13] | -0.06 [-0.23, 0.12] |
| Acquisition CS+ Learning Rate (US-) | -0.10 [-0.25, 0.07] | -0.09 [-0.23, 0.08] | -0.06 [-0.22, 0.12] | -0.04 [-0.21, 0.13] |
| Acquisition CS- Learning Rate | **-0.22 [-0.37, -0.05]** | **-0.23 [-0.37, -0.06]** | -0.14 [-0.30, 0.02] | -0.15 [-0.30, 0.01] |
| Extinction CS+ Learning Rate | **-0.21 [-0.37, -0.05]** | **-0.21 [-0.36, -0.05]** | **-0.23 [-0.37, -0.06]** | **-0.22 [-0.37, -0.06]** |
| Extinction CS- Learning Rate | -0.07 [-0.23, 0.09] | -0.06 [-0.22, 0.10] | -0.10 [-0.26, 0.07] | -0.08 [-0.24, 0.08] |
| Lapse | -0.06 [-0.21, 0.10] | -0.03 [-0.20, 0.14] | -0.16 [-0.32, 0.00] | -0.13 [-0.28, 0.05] |
| ***Bold ~ p < 0.05*** | | | | |
| *Regular = standard Spearman correlation; Partial = controlling for scream unpleasantness* | | | | |
| *GAD-7 - Generalized Anxiety Disorder seven item scale; PHQ-8 - Patient Health Questionnaire eight item scale* | | | | |
| *Scream unpleasantness correlations: GAD-7 ρ = 0.11 (p = 0.191), PHQ-8 ρ = 0.14 (p = 0.084)* | | | | |

Partial Spearman correlation, correcting for US unpleasantness rating in strict excluded dataset (n=88). This did remove the association between the lapse parameter and PHQ-8.

|  | GAD-7 | | PHQ-8 | |
| --- | --- | --- | --- | --- |
| Parameter | Regular ρ [95% CI] | Partial ρ [95% CI] | Regular ρ [95% CI] | Partial ρ [95% CI] |
| Acquisition CS+ Learning Rate (US+) | -0.15 [-0.36, 0.06] | -0.17 [-0.37, 0.06] | -0.01 [-0.22, 0.20] | -0.02 [-0.24, 0.20] |
| Acquisition CS+ Learning Rate (US-) | **-0.30 [-0.49, -0.08]** | **-0.29 [-0.49, -0.06]** | -0.20 [-0.38, 0.02] | -0.19 [-0.39, 0.02] |
| Acquisition CS- Learning Rate | **-0.32 [-0.48, -0.13]** | **-0.32 [-0.49, -0.14]** | -0.14 [-0.34, 0.07] | -0.14 [-0.33, 0.05] |
| Extinction CS+ Learning Rate | **-0.33 [-0.51, -0.14]** | **-0.33 [-0.52, -0.12]** | **-0.26 [-0.43, -0.03]** | **-0.25 [-0.43, -0.04]** |
| Extinction CS- Learning Rate | -0.15 [-0.34, 0.08] | -0.14 [-0.33, 0.08] | -0.13 [-0.35, 0.10] | -0.12 [-0.34, 0.09] |
| Lapse | -0.13 [-0.36, 0.07] | -0.12 [-0.32, 0.10] | **-0.22 [-0.41, 0.00]** | -0.20 [-0.38, 0.02] |
| ***Bold ~ p < 0.05*** | | | | |
| *Regular = standard Spearman correlation; Partial = controlling for scream unpleasantness* | | | | |
| *GAD-7 - Generalized Anxiety Disorder seven item scale; PHQ-8 - Patient Health Questionnaire eight item scale* | | | | |
| *Scream unpleasantness correlations: GAD-7 ρ = 0.09 (p = 0.431), PHQ-8 ρ = 0.11 (p = 0.289)* | | | | |

Residuals analysis

|  | Medium Criteria (n=145) | | | | | Strict Criteria (n=88) | | | | |
| --- | --- | --- | --- | --- | --- | --- | --- | --- | --- | --- |
| Parameter | GAD-7 | PHQ-8 | GAD-7 residual | PHQ-8 residual | Shared variance | GAD-7 | PHQ-8 | GAD-7 residual | PHQ-8 residual | Shared variance |
| Acquisition CS+ Learning Rate (US+) | -0.07 [-0.23, 0.10] | -0.03 [-0.20, 0.13] | -0.03 [-0.21, 0.15] | 0.02 [-0.13, 0.18] | -0.05 [-0.22, 0.11] | -0.15 [-0.36, 0.05] | -0.01 [-0.20, 0.21] | -0.16 [-0.39, 0.08] | 0.13 [-0.10, 0.34] | -0.07 [-0.27, 0.16] |
| Acquisition CS+ Learning Rate (US-) | -0.10 [-0.26, 0.06] | -0.06 [-0.22, 0.10] | -0.06 [-0.24, 0.12] | -0.01 [-0.17, 0.16] | -0.08 [-0.23, 0.09] | **-0.30 [-0.50, -0.08]** | -0.20 [-0.40, 0.03] | -0.21 [-0.41, 0.02] | 0.03 [-0.19, 0.25] | **-0.26 [-0.45, -0.05]** |
| Acquisition CS- Learning Rate | **-0.22 [-0.37, -0.06]** | -0.14 [-0.29, 0.02] | -0.14 [-0.29, 0.04] | 0.01 [-0.16, 0.19] | **-0.18 [-0.34, -0.02]** | **-0.32 [-0.50, -0.11]** | -0.14 [-0.33, 0.06] | **-0.28 [-0.46, -0.07]** | 0.16 [-0.04, 0.36] | **-0.22 [-0.41, -0.02]** |
| Extinction CS+ Learning Rate | **-0.21 [-0.39, -0.05]** | **-0.23 [-0.38, -0.06]** | -0.06 [-0.22, 0.10] | -0.09 [-0.26, 0.08] | **-0.24 [-0.39, -0.09]** | **-0.33 [-0.51, -0.13]** | **-0.26 [-0.44, -0.04]** | -0.17 [-0.38, 0.05] | -0.03 [-0.25, 0.19] | **-0.31 [-0.48, -0.12]** |
| Extinction CS- Learning Rate | -0.07 [-0.21, 0.09] | -0.10 [-0.26, 0.07] | 0.06 [-0.10, 0.21] | -0.09 [-0.24, 0.07] | -0.10 [-0.25, 0.07] | -0.15 [-0.36, 0.07] | -0.13 [-0.35, 0.09] | 0.05 [-0.16, 0.25] | -0.08 [-0.30, 0.13] | -0.16 [-0.35, 0.04] |
| ***Bold ~ p < 0.05*** | | | | | | | | | | |
| *Values show Spearman correlation ρ [95% CI]. GAD-7 and PHQ-8 correlations ~ Medium ρ = 0.71, Strict ρ = 0.74* | | | | | | | | | | |
| *GAD-7 - Generalized Anxiety Disorder seven item scale; PHQ-8 - Patient Health Questionnaire eight item scale* | | | | | | | | | | |

## Median split


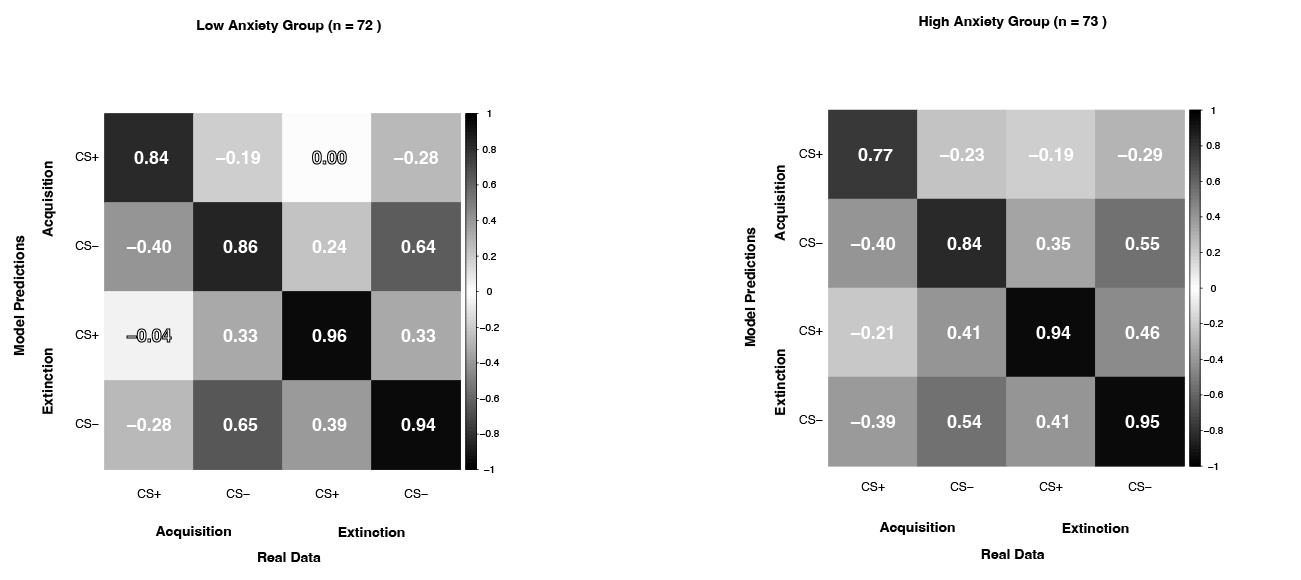

Supplement: Supplementary Materials. — Supplementary Methods & Supplementary Results. [file cpsy-10-1-138-s1.zip › cpsy-138_kerr/68b6a9abb0c8e.docx]
