# Supplementary material for: Computational Modelling Reveals Slower Safety Learning and Threat Extinction are Associated With Higher Anxiety Severity in Remote Fear Conditioning
Source: Comput Psychiatr. 2026 Jan 21;10(1):18–35. doi: 10.5334/cpsy.138 (PMC12829443; doi:10.5334/cpsy.138)

Supplementary Methods

Counter-factual model specification

The Rescorla-Wagner model was expanded with an additional learning rate, to model the counter-factual value updating observed from CS+ trials on to subsequent CS- trials, in acquisition phase. This updating was seen to be bi-directional, both within and across participants, i.e. the prediction error from a CS+ trial could generate either a negative or positive update on the next CS- trial, increasing or decreasing the US Expectancy Rating (ER) respectively.

Building on model 2d from the main analysis, which had two learning rates, LR^{CS+} and LR^{CS-}, an additional learning rate, $LR^{fictive}$ was applied to all CS+ trials. Unlike the other learning rates, this was in the interval [-1, 1], to allow for negative and positive value updating. This process 'transfers' the prediction error from CS+ to CS- trials.

$$V_{t+1}^{CS+} = V_t^{CS+} + (US - V_t^{CS+}) \cdot LR^{CS+}$$

$$V_{t+1}^{CS-} = V_t^{CS-} + (US - V_t^{CS+}) \cdot LR^{fictive}$$

To prevent VCS- exceeding the interval [0,1], it was passed through maximum and minimum functions prior to being transformed into the ordinal vector of probabilities used to estimate ER. This model is referred to as Counter-Factual Model 1.

$$V^{CS-} = min\big(1, max(0, V^{CS-})\big)$$

To examine the possibility of the strength of counterfactual updating diminishing over trials, a further model was tested with a decay rate applied to $LR^{fictive}$. This reduced the strength of the counterfactual update as a function of trial number. This model is referred to as Counter-Factual Model 2.

$$LR^{fictive} = LR^{fictive} \cdot e^{(-decay \cdot t)}$$

Two further models were tested, which counterfactually updated only on surprising trials, i.e. the three unreinforced CS+ trials, without and with the decay rate applied. These models are referred to as Counter-Factual Model 3 and Counter-Factual Model 4, respectively.

Simulations and parameter recovery were performed on these four models. Results of parameter recovery are detailed in the section Supplementary Results.

Supplementary Results

Exclusions

Venn diagrams of exclusions indicate the numbers excluded based on one or more (intersections) criteria. For instance, many participants both reduced volume and removed headphones.

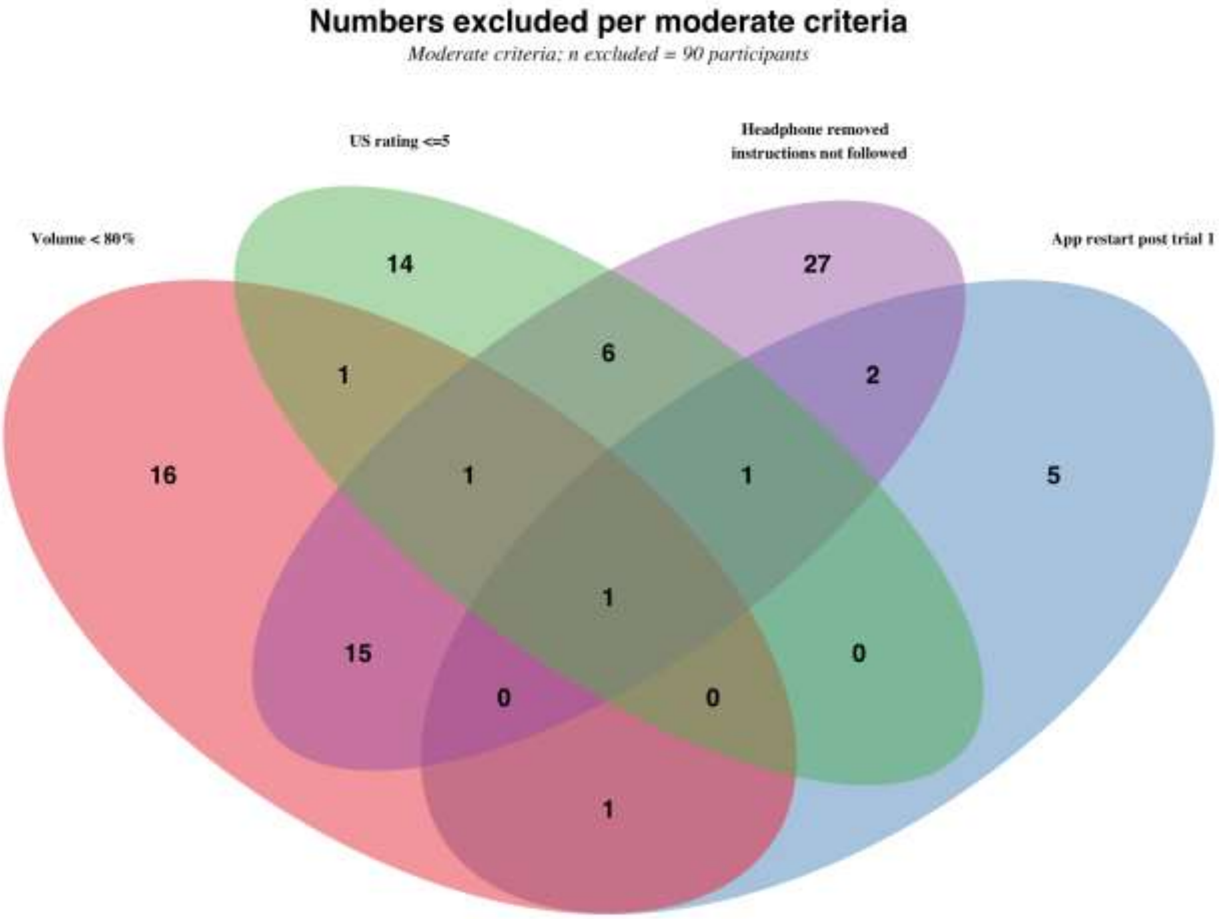

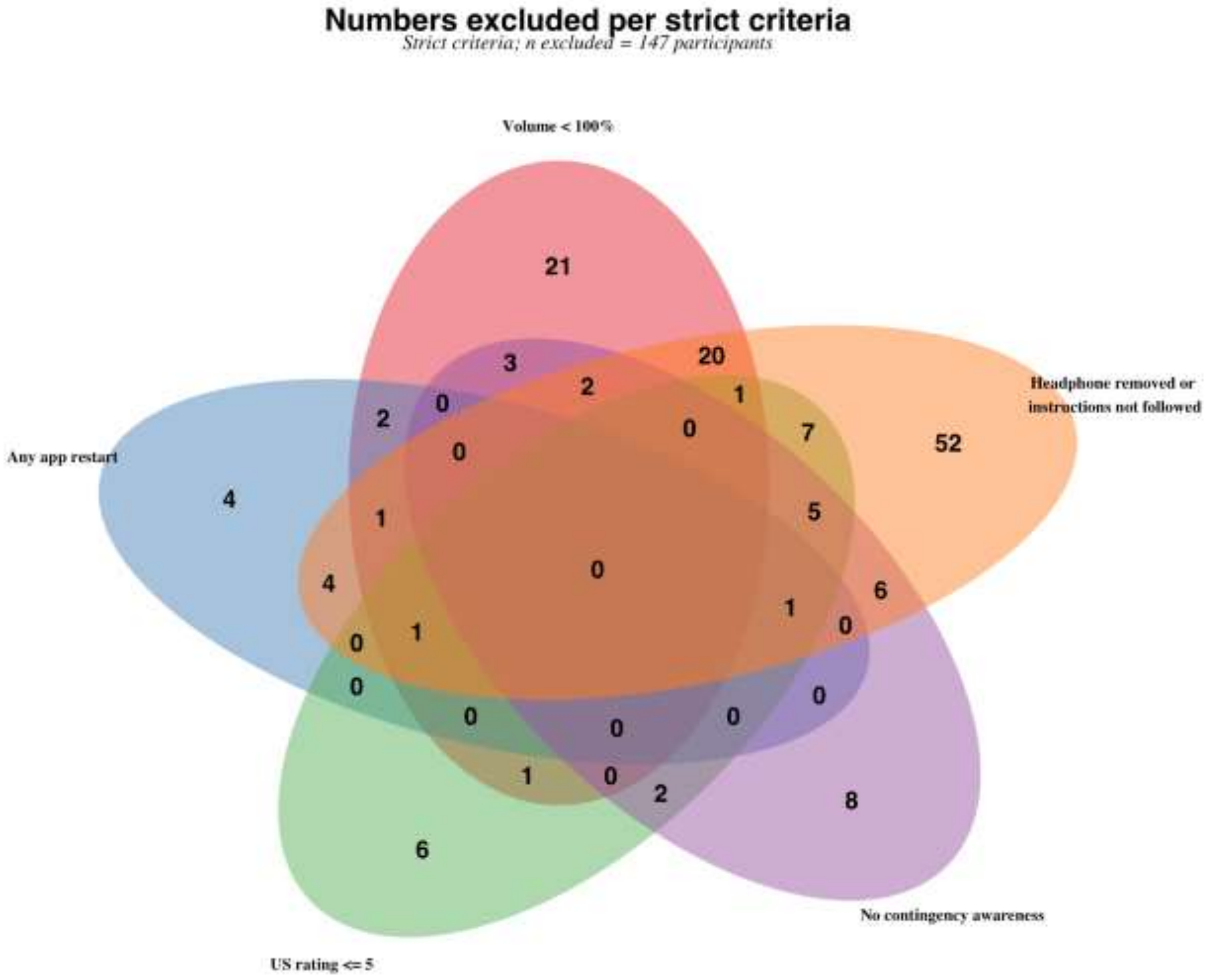
Numbers excluded per strict criteria
Strict criteria; n excluded = 147 participants
Volume < 100%
Headphone removed or instructions not followed
Any app restart
No contingency awareness
US rating <= 5
21
3
20
2
1
2
0
0
7
52
0
4
1
5
0
4
6
1
0
1
0
0
0
0
0
0
0
1
0
2
8
6

Parameter Recovery

For each model subtype, three ranges of parameters were simulated and tested. Broad simulated parameters covering the entire parameter space (i.e. 0 to 1 for a learning rate). Low simulated parameters biased to the lower end of the space (i.e. 0 to 0.3 for a learning rate). High simulated parameters biased to the upper end of the space (i.e. 0.7 to 1 for a learning rate).

The simulated data was drawn from the CS patterns used by real participants. 145 participants were simulated each time. Recovery was assessed as successful if the known value is contained within the 95CI of the parameter estimate. Where estimates are broad and cover most of the parameter space, this metric is less useful (this is best visualised in the corresponding plots, and tends to occur for parameters based on single trials like the start parameters or CS Jump). Therefore, correlation with the known value was also calculated.

Each cell in the table shows Recovery%/Correlation. The aim was for >95% recovery for learning rate parameters.

Model 1

Model	Subtype	Parameter_Range	lr	lapse	start_csp	start_csm	start_csm_jump
1	a	broad	95.2%/0.98	95.9%/0.89	-/-	-/-	-/-
1	b	broad	92.4%/0.96	94.5%/0.88	93.8%/0.67	98.6%/0.76	-/-
1	c	broad	94.5%/0.98	95.9%/0.88	-/-	-/-	95.9%/0.65
1	d	broad	95.9%/0.98	98.6%/0.89	91.7%/0.73	97.2%/0.81	97.2%/0.79
1	a	high	93.8%/0.98	93.8%/0.87	-/-	-/-	-/-
1	b	high	95.9%/0.97	96.6%/0.90	95.9%/0.51	99.3%/0.68	-/-
1	c	high	95.2%/0.97	96.6%/0.89	-/-	-/-	95.2%/0.55

1	d	high	96.6%/0.97	95.9%/0.90	97.9%/0.64	95.9%/0.70	96.6%/0.63
1	a	low	96.6%/0.98	95.2%/0.87	-/-	-/-	-/-
1	b	low	95.2%/0.99	95.9%/0.90	92.4%/0.70	97.9%/0.86	-/-
1	c	low	94.5%/0.99	97.9%/0.87	-/-	-/-	93.8%/0.79
1	d	low	91%/0.97	95.9%/0.88	97.9%/0.83	95.9%/0.88	93.8%/0.79

Model 2

Model	Subtype	Parameter_ Range	lr_csp	lr_csm	lapse	start_c sp	start_c sm	start_csm_ jump
2	a	broad	97.9%/ 0.98	92.4%/ 0.85	94.5%/ 0.87	-/-	-/-	-/-
2	b	broad	95.9%/ 0.97	95.2%/ 0.77	96.6%/ 0.89	95.9%/ 0.76	92.4%/ 0.63	-/-
2	c	broad	93.1%/ 0.97	97.2%/ 0.92	95.2%/ 0.89	-/-	-/-	92.4%/0.73
2	d	broad	94.5%/ 0.98	94.5%/ 0.89	95.9%/ 0.88	94.5%/ 0.69	95.2%/ 0.68	94.5%/0.74
2	a	high	95.9%/ 0.96	93.8%/ 0.75	97.9%/ 0.89	-/-	-/-	-/-
2	b	high	95.2%/ 0.95	95.2%/ 0.77	94.5%/ 0.85	94.5%/ 0.65	98.6%/ 0.62	-/-
2	c	high	96.6%/ 0.96	86.9%/ 0.78	96.6%/ 0.88	-/-	-/-	95.2%/0.69
2	d	high	98.6%/ 0.96	96.6%/ 0.77	93.8%/ 0.87	93.8%/ 0.64	93.8%/ 0.56	95.9%/0.69
2	a	low	93.8%/ 0.98	95.9%/ 0.93	94.5%/ 0.91	-/-	-/-	-/-
2	b	low	95.2%/ 0.98	93.8%/ 0.83	93.1%/ 0.88	93.8%/ 0.77	94.5%/ 0.60	-/-
2	c	low	95.2%/ 0.97	96.6%/ 0.96	95.2%/ 0.90	-/-	-/-	95.9%/0.82
2	d	low	96.6%/ 0.98	93.8%/ 0.92	93.1%/ 0.88	94.5%/ 0.75	95.9%/ 0.76	95.2%/0.83

Model 3

Model	Subtype	Parameter_Range	lr_csp_acq	lr_csp_ext	lr_csm	lapse	start_csp	start_csm	start_csm_jump
3	a	broad	96.6%/0.96	91%/0.85	94.5%/0.87	91%/0.88	-/-	-/-	-/-
3	b	broad	96.6%/0.93	96.6%/0.90	95.9%/0.80	97.2%/0.91	93.8%/0.69	95.9%/0.71	-/-
3	c	broad	92.4%/0.94	95.2%/0.90	96.6%/0.88	96.6%/0.92	-/-	-/-	97.2%/0.74
3	d	broad	93.1%/0.92	93.8%/0.84	98.6%/0.88	96.6%/0.90	95.2%/0.74	97.2%/0.68	95.2%/0.61
3	a	high	93.1%/0.92	92.4%/0.62	96.6%/0.82	93.1%/0.88	-/-	-/-	-/-
3	b	high	93.1%/0.88	96.6%/0.77	96.6%/0.56	94.5%/0.89	96.6%/0.64	93.8%/0.51	-/-
3	c	high	94.5%/0.91	95.9%/0.76	93.8%/0.84	96.6%/0.91	-/-	-/-	96.6%/0.61
3	d	high	95.2%/0.91	95.2%/0.69	97.2%/0.80	95.2%/0.89	97.9%/0.62	93.8%/0.54	95.9%/0.72
3	a	low	96.6%/0.91	95.9%/0.95	95.2%/0.92	94.5%/0.89	-/-	-/-	-/-
3	b	low	96.6%/0.86	97.2%/0.95	97.2%/0.81	97.2%/0.89	96.6%/0.77	93.8%/0.70	-/-
3	c	low	97.2%/0.89	95.2%/0.95	95.2%/0.96	96.6%/0.92	-/-	-/-	93.8%/0.73
3	d	low	91.7%/0.86	96.6%/0.95	97.9%/0.90	97.2%/0.91	95.2%/0.73	95.9%/0.81	92.4%/0.78

Model 4

Model	Subtype	Parameter_Range	lr_csp_acq	lr_csp_ext	lr_csm_acq	lr_csm_ext	lapse	start_csp	start_csm	start_csm_jump
4	a	broad	95.9%/0.94	97.2%/0.87	95.2%/0.83	99.3%/0.03	97.2%/0.91	-/-	-/-	-/-
4	b	broad	95.9%/0.93	93.8%/0.86	94.5%/0.74	100%/-0.06	99.3%/0.90	96.6%/0.70	94.5%/0.65	-/-
4	c	broad	95.2%/0.96	94.5%/0.90	97.2%/0.89	97.2%/0.76	95.9%/0.91	-/-	-/-	96.6%/0.65
4	d	broad	93.1%/0.92	95.2%/0.88	94.5%/0.76	95.2%/0.75	97.2%/0.89	94.5%/0.68	95.2%/0.64	95.9%/0.65
4	a	high	96.6%/0.95	94.5%/0.63	96.6%/0.82	97.2%/0.12	97.9%/0.91	-/-	-/-	-/-
4	b	high	94.5%/0.89	91%/0.65	99.3%/0.69	99.3%/-0.06	97.9%/0.90	95.2%/0.64	97.9%/0.53	-/-
4	c	high	94.5%/0.94	96.6%/0.72	96.6%/0.82	96.6%/0.65	95.9%/0.91	-/-	-/-	91.7%/0.44
4	d	high	97.2%/0.91	95.2%/0.67	98.6%/0.70	93.8%/0.60	95.2%/0.89	94.5%/0.62	96.6%/0.55	95.2%/0.64
4	a	low	95.2%/0.95	96.6%/0.95	93.1%/0.92	97.2%/0.22	95.2%/0.90	-/-	-/-	-/-
4	b	low	97.9%/0.93	95.9%/0.94	95.9%/0.80	96.6%/0.24	97.9%/0.90	97.2%/0.75	96.6%/0.73	-/-

4	c	low	96.6%/0.91	95.9%/0.93	95.9%/0.94	94.5%/0.83	93.8%/0.90	-/-	-/-	95.2%/0.78
4	d	low	93.8%/0.90	97.2%/0.94	96.6%/0.79	93.8%/0.82	97.2%/0.90	95.9%/0.70	92.4%/0.71	95.2%/0.71

Model 5

Model	Subtype	Parameter_Range	lr_pos	lr_neg	lapse	start_csp	start_csm	start_csm_jump
5	a	broad	97.2%/ 0.96	94.5%/ 0.95	94.5%/ 0.87	-/-	-/-	-/-
5	b	broad	97.2%/ 0.96	93.1%/ 0.94	96.6%/ 0.89	96.6%/ 0.66	92.4%/ 0.70	-/-
5	c	broad	94.5%/ 0.96	96.6%/ 0.96	95.9%/ 0.90	-/-	-/-	93.1%/0.76
5	d	broad	95.2%/ 0.95	95.9%/ 0.97	95.2%/ 0.89	92.4%/ 0.63	93.8%/ 0.74	93.8%/0.81
5	a	high	96.6%/ 0.93	95.2%/ 0.92	95.2%/ 0.88	-/-	-/-	-/-
5	b	high	96.6%/ 0.93	96.6%/ 0.92	92.4%/ 0.84	92.4%/ 0.65	95.2%/ 0.63	-/-
5	c	high	99.3%/ 0.94	94.5%/ 0.95	95.9%/ 0.88	-/-	-/-	95.2%/0.70
5	d	high	97.9%/ 0.93	96.6%/ 0.94	94.5%/ 0.84	93.1%/ 0.65	95.9%/ 0.60	97.2%/0.76
5	a	low	94.5%/ 0.96	92.4%/ 0.98	94.5%/ 0.90	-/-	-/-	-/-
5	b	low	93.1%/ 0.93	95.9%/ 0.97	93.1%/ 0.86	93.1%/ 0.67	93.1%/ 0.76	-/-
5	c	low	94.5%/ 0.94	93.8%/ 0.98	95.2%/ 0.90	-/-	-/-	94.5%/0.84
5	d	low	92.4%/ 0.92	95.9%/ 0.97	95.2%/ 0.88	93.8%/ 0.68	94.5%/ 0.81	94.5%/0.89

Model 6

Model	Subtype	Parameter_Range	lr_csp_pos	lr_csp_neg	lr_csm	lapse	start_csp	start_csm	start_csm_jump
6	a	broad	95.9%/ 0.95	93.8%/ 0.91	93.8%/ 0.87	93.1%/ 0.87	-/-	-/-	-/-
6	b	broad	93.8%/ 0.93	96.6%/ 0.94	95.9%/ 0.80	98.6%/ 0.92	93.1%/ 0.68	97.2%/ 0.70	-/-
6	c	broad	94.5%/ 0.96	97.9%/ 0.95	97.2%/ 0.88	96.6%/ 0.93	-/-	-/-	97.2%/0.74
6	d	broad	95.2%/ 0.95	92.4%/ 0.92	97.9%/ 0.88	95.9%/ 0.90	96.6%/ 0.73	97.9%/ 0.68	95.9%/0.62
6	a	high	95.2%/ 0.85	97.2%/ 0.90	97.2%/ 0.83	93.8%/ 0.90	-/-	-/-	-/-
6	b	high	95.2%/ 0.88	94.5%/ 0.89	95.9%/ 0.56	93.1%/ 0.89	95.9%/ 0.60	95.2%/ 0.51	-/-
6	c	high	95.2%/ 0.91	95.9%/ 0.92	95.2%/ 0.84	95.9%/ 0.91	-/-	-/-	96.6%/0.60
6	d	high	94.5%/ 0.92	95.2%/ 0.87	97.9%/ 0.80	96.6%/ 0.90	97.2%/ 0.58	93.8%/ 0.55	96.6%/0.72
6	a	low	95.2%/ 0.94	93.8%/ 0.93	95.9%/ 0.92	95.2%/ 0.89	-/-	-/-	-/-
6	b	low	95.2%/ 0.90	94.5%/ 0.94	96.6%/ 0.81	95.2%/ 0.89	97.9%/ 0.73	95.2%/ 0.71	-/-
6	c	low	95.2%/ 0.93	93.8%/ 0.96	95.9%/ 0.96	97.2%/ 0.92	-/-	-/-	93.1%/0.73
6	d	low	94.5%/ 0.90	95.9%/ 0.95	95.9%/ 0.90	96.6%/ 0.91	97.9%/ 0.67	97.2%/ 0.81	93.8%/0.78

Model 7

Model	Subtype	Parameter_Range	lr_csp_acq_us1	lr_csp_acq_us0	lr_csp_ext	lr_csm_acq	lr_csm_ext	lapse	start_csp	start_csm	start_csm_jump
7	a	broad	97.2%/0.94	96.6%/0.84	96.6%/0.87	93.1%/0.84	100%/0.19	97.9%/0.91	-/-	-/-	-/-
7	b	broad	94.5%/0.94	96.6%/0.84	98.6%/0.85	97.9%/0.81	100%/0.03	95.9%/0.89	92.4%/0.56	96.6%/0.65	-/-
7	c	broad	96.6%/0.95	97.9%/0.72	97.2%/0.86	97.9%/0.91	92.4%/0.77	97.9%/0.90	-/-	-/-	91.7%/0.58
7	d	broad	95.9%/0.94	97.2%/0.76	98.6%/0.85	99.3%/0.80	96.6%/0.84	97.2%/0.90	94.5%/0.56	97.9%/0.71	93.1%/0.58
7	a	high	95.9%/0.94	93.1%/0.76	95.9%/0.69	95.2%/0.83	98.6%/-0.11	95.2%/0.92	-/-	-/-	-/-
7	b	high	94.5%/0.88	97.2%/0.75	95.2%/0.63	91.7%/0.60	97.9%/-0.16	98.6%/0.90	96.6%/0.64	95.9%/0.46	-/-
7	c	high	95.9%/0.93	93.1%/0.72	95.9%/0.77	96.6%/0.75	95.2%/0.65	95.9%/0.88	-/-	-/-	97.9%/0.56
7	d	high	95.2%/0.92	95.2%/0.77	93.1%/0.71	93.8%/0.67	93.8%/0.70	98.6%/0.90	93.1%/0.57	96.6%/0.72	92.4%/0.63
7	a	low	94.5%/0.92	96.6%/0.68	95.9%/0.93	91%/0.93	97.9%/0.27	95.9%/0.89	-/-	-/-	-/-
7	b	low	93.1%/0.91	97.9%/0.74	97.2%/0.94	92.4%/0.79	100%/0.27	94.5%/0.90	95.2%/0.66	95.2%/0.64	-/-

7	c	low	97.2%/0.93	95.2%/0.63	93.1%/0.93	93.1%/0.92	95.9%/0.84	93.8%/0.88	-/-	-/-	97.2%/0.68
7	d	low	95.9%/0.90	92.4%/0.66	93.8%/0.93	97.2%/0.83	92.4%/0.86	96.6%/0.89	95.2%/0.73	98.6%/0.74	95.9%/0.74

Counter-factual model 1 (fictive update on all CS+ trials)

param_range	lr_csm	lr_csp	lr_fic	decay_rate	lapse	start_csp	start_csm	start_csm_jump
broad	95.9%/0.97	93.8%/1.00	96.6%/0.96	-/-	100%/0.31	95.2%/0.98	93.8%/0.94	95.2%/0.96
high	93.8%/0.98	91.7%/0.99	93.1%/0.99	-/-	98.6%/0.30	95.2%/0.98	91.7%/0.93	96.6%/0.96
low	94.5%/0.93	91.7%/1.00	95.9%/0.75	-/-	99.3%/0.42	95.9%/0.98	97.2%/0.94	97.2%/0.98

Counter-factual model 2 (fictive update on all CS+ trials & decay on fictive learning rate)

param_range	lr_csm	lr_csp	lr_fic	decay_rate	lapse	start_csp	start_csm	start_csm_jump
broad	95.2%/0.96	94.5%/1.00	98.6%/0.62	97.9%/0.47	98.6%/0.19	94.5%/0.98	97.2%/0.94	93.8%/0.97
high	96.6%/0.96	93.1%/0.99	99.3%/0.49	98.6%/0.42	100%/0.29	95.9%/0.98	95.9%/0.96	95.2%/0.98
low	92.4%/0.90	97.2%/1.00	100%/0.50	97.2%/0.23	98.6%/0.59	93.1%/0.98	93.1%/0.93	96.6%/0.97

Counter-factual model 3 (fictive update only on CS+US-trials)

param_range	lr_csm	lr_csp	lr_fic	decay_rate	lapse	start_csp	start_csm	start_csm_jump
broad	96.6%/0.98	94.5%/1.00	94.5%/0.94	-/-	97.9%/0.24	94.5%/0.98	98.6%/0.98	97.9%/0.95
high	94.5%/0.99	95.2%/0.99	92.4%/0.98	-/-	99.3%/0.41	89.7%/0.97	95.2%/0.99	92.4%/0.87
low	95.2%/0.92	91%/1.00	97.9%/0.67	-/-	99.3%/0.43	93.8%/0.99	94.5%/0.95	95.9%/0.96

Counter-factual model 4 (fictive update only on CS+US- trials & decay on fictive learning rate)

param_range	lr_csm	lr_csp	lr_fic	decay_rate	lapse	start_csp	start_csm	start_csm_jump
broad	95.2%/0.97	93.8%/1.00	100%/0.53	97.9%/0.43	98.6%/0.20	95.2%/0.98	98.6%/0.98	94.5%/0.96
high	96.6%/0.98	93.8%/0.99	95.9%/0.25	95.2%/0.41	100%/0.35	95.9%/0.98	94.5%/0.98	95.9%/0.98
low	94.5%/0.87	98.6%/1.00	100%/0.48	97.9%/0.21	99.3%/0.58	93.8%/0.98	93.8%/0.95	97.2%/0.97

Prior Predictive Checks

Prior predictive check for model with single learning rate and no extra fitting parameters (model 1a). This fails to provide model space for participant behaviour in some sections

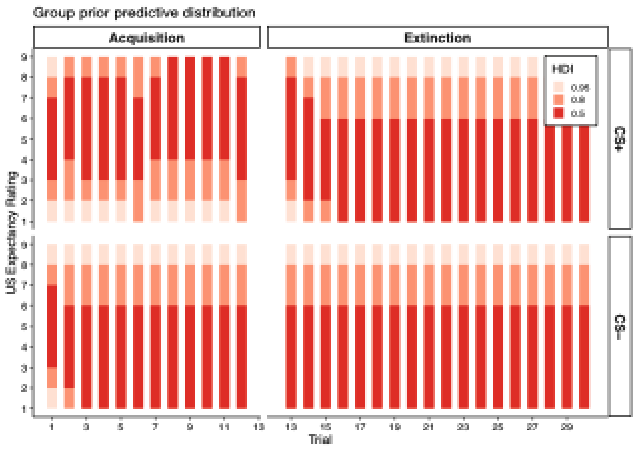

Prior predictive check for the winning model, with five learning rate parameters and three fitting parameters (model 7d). This provides model space for participant data to be modelled accurately.

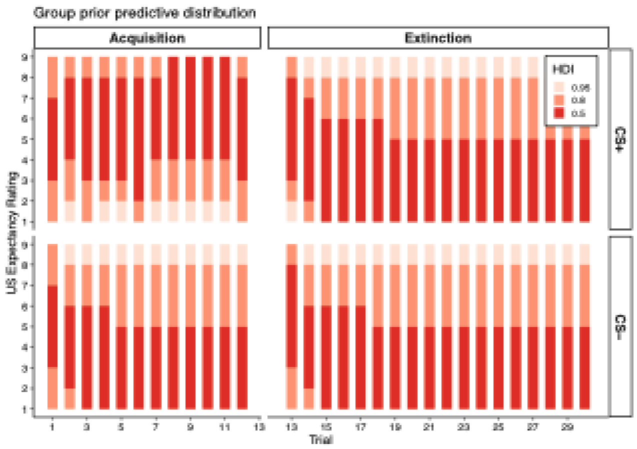

Model Comparison

Bar chart indicating LOOIC for each model. Black lines represent the best fitting in each model class (between variants a, b, c, and d). 7d was the best fitting model across all models, with lowest LOOIC.

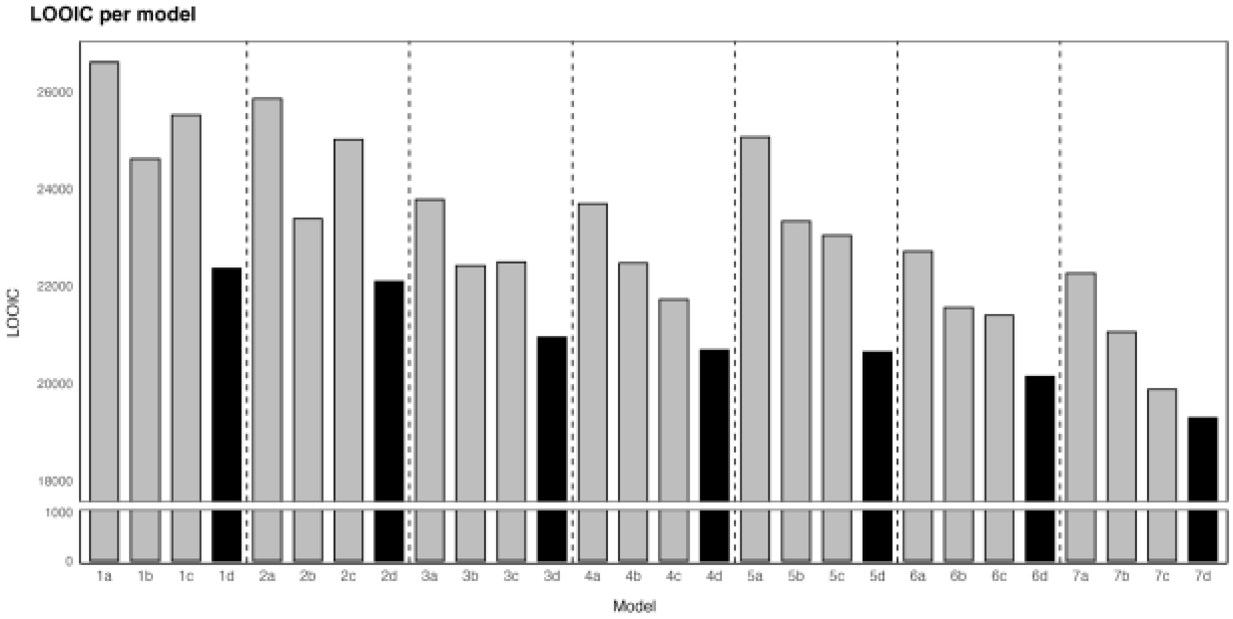

Overall model comparison table. Bold indicates the best metric i.e. highest likelihood or pseudo r2, or lowest LOOIC.

Model Comparison Results											
Model #	Total Params	LR Params	Start Params	Jump Params	Log Likelihood	LOOIC	WAIC	BIC	Integrated BIC	Pseudo R^2	LOO
7d	1323	5	2	1	**−1,787,621.40**	**19,291.66**	**19,654.71**	26,294.50	26,046.56	**0.52**	**−9,645.83**
7c	1029	5	0	1	−1,865,452.84	19,877.80	20,223.85	25,202.08	25,077.75	0.50	−9,938.90
7b	1176	5	2	0	−1,946,379.09	21,064.23	21,231.79	26,946.71	26,777.30	0.48	−10,532.12
7a	882	5	0	0	−2,069,167.63	22,263.33	22,965.95	26,303.87	26,156.55	0.45	−11,131.67
6d	1029	3	2	1	−1,880,168.45	20,144.12	20,170.68	25,349.24	25,243.10	0.50	−10,072.06
6c	735	3	0	1	−2,008,271.16	21,409.82	21,573.19	**24,759.54**	**24,657.82**	0.47	−10,704.91
6b	882	3	2	0	−2,014,910.47	21,558.29	21,627.16	25,761.30	25,629.17	0.46	−10,779.15
6a	588	3	0	0	−2,149,653.85	22,717.22	22,839.47	25,238.00	25,135.83	0.43	−11,358.61
5d	882	2	2	1	−1,929,506.71	20,649.24	20,792.12	24,907.26	24,767.55	0.49	−10,324.62
5c	588	2	0	1	−2,157,918.57	23,047.71	23,263.48	25,320.65	25,232.15	0.43	−11,523.85
5b	735	2	2	0	−2,152,127.27	23,339.66	24,578.08	26,198.10	26,049.61	0.43	−11,669.83
5a	441	2	0	0	−2,340,527.31	25,067.09	25,792.11	26,211.37	26,131.14	0.38	−12,533.54
4d	1176	4	2	1	−1,923,890.77	20,687.78	20,984.37	26,721.83	26,544.81	0.49	−10,343.89
4c	882	4	0	1	−2,048,224.50	21,725.33	22,243.53	26,094.44	25,946.72	0.45	−10,862.66
4b	1029	4	2	0	−2,080,351.25	22,474.56	23,048.15	27,351.07	27,210.59	0.45	−11,237.28
4a	735	4	0	0	−2,228,597.62	23,699.64	24,454.17	26,962.80	26,839.54	0.41	−11,849.82
3d	1029	3	2	1	−1,950,932.94	20,954.90	21,407.94	26,056.89	25,881.01	0.48	−10,477.45
3c	735	3	0	1	−2,114,543.96	22,495.57	22,979.96	25,822.27	25,712.78	0.44	−11,247.79
3b	882	3	2	0	−2,093,060.27	22,421.72	22,733.13	26,542.79	26,370.99	0.44	−11,210.86
3a	588	3	0	0	−2,253,001.89	23,784.89	24,669.18	26,271.48	26,166.56	0.40	−11,892.44
2d	882	2	2	1	−2,049,759.95	22,094.50	23,211.88	26,109.79	25,974.34	0.45	−11,047.25
2c	588	2	0	1	−2,313,063.62	25,017.74	26,390.70	26,872.10	26,731.16	0.38	−12,508.87
2b	735	2	2	0	−2,180,016.38	23,391.10	24,422.11	26,476.99	26,307.36	0.42	−11,695.55
2a	441	2	0	0	−2,425,821.63	25,860.48	27,191.23	27,064.31	26,950.94	0.35	−12,930.24
1d	735	1	2	1	−2,099,620.56	22,365.42	23,666.74	25,673.03	25,500.41	0.44	−11,182.71
1c	441	1	0	1	−2,399,215.47	25,520.28	25,971.31	26,798.25	26,728.29	0.36	−12,760.14
1b	588	1	2	0	−2,296,692.74	24,614.97	25,852.89	26,708.39	26,578.54	0.39	−12,307.49
1a	294	1	0	0	−2,533,454.26	26,605.49	27,164.61	27,205.27	27,130.54	0.33	−13,302.75

Model comparison table for acquisition CS+ trials.

Model Comparison - Acquisition CS+											
Model #	Total Params	LR Params	Start Params	Jump Params	Log Likelihood	LOOIC	WAIC	BIC	Integrated BIC	Pseudo R^2	LOO
7d	1323	5	2	1	**−441,612.49**	**4,861.90**	**4,880.59**	11,000.34	10,889.48	**0.41**	**−2,430.95**
7c	1029	5	0	1	−478,722.07	5,107.06	5,121.91	9,908.28	9,843.50	0.36	−2,553.53
7b	1176	5	2	0	−441,853.27	4,868.98	4,905.73	10,271.17	10,169.08	0.41	−2,434.49
7a	882	5	0	0	−481,873.51	5,140.86	5,152.89	9,208.21	9,130.34	0.36	−2,570.43
6d	1029	3	2	1	−489,223.41	5,298.63	5,320.05	10,013.29	9,932.44	0.35	−2,649.32
6c	735	3	0	1	−528,455.59	5,577.42	5,598.24	8,942.46	8,868.23	0.30	−2,788.71
6b	882	3	2	0	−488,323.68	5,269.10	5,288.59	9,272.72	9,154.25	0.35	−2,634.55
6a	588	3	0	0	−529,266.19	5,558.28	5,556.16	8,218.98	8,157.42	0.30	−2,779.14
5d	882	2	2	1	−489,526.59	5,315.32	5,385.97	9,284.75	9,194.50	0.35	−2,657.66
5c	588	2	0	1	−532,670.22	5,589.09	5,607.42	8,253.02	8,197.34	0.29	−2,794.55
5b	735	2	2	0	−502,961.92	5,470.50	5,611.56	8,687.52	8,584.15	0.33	−2,735.25
5a	441	2	0	0	−547,948.30	5,757.55	5,844.72	**7,674.22**	**7,589.95**	0.27	−2,878.78
4d	1176	4	2	1	−546,769.66	5,960.78	6,178.74	11,320.34	11,210.50	0.27	−2,980.39
4c	882	4	0	1	−609,162.51	6,413.23	6,454.15	10,481.10	10,399.87	0.19	−3,206.61
4b	1029	4	2	0	−542,917.24	5,899.10	6,103.90	10,550.23	10,440.61	0.28	−2,949.55
4a	735	4	0	0	−598,549.50	6,260.41	6,262.61	9,643.39	9,554.96	0.20	−3,130.20
3d	1029	3	2	1	−545,601.64	5,950.26	6,160.58	10,577.08	10,495.52	0.27	−2,975.13
3c	735	3	0	1	−605,297.90	6,355.43	6,379.39	9,710.88	9,629.90	0.19	−3,177.72
3b	882	3	2	0	−545,571.90	5,945.93	6,138.84	9,845.20	9,754.27	0.27	−2,972.97
3a	588	3	0	0	−599,683.60	6,273.33	6,294.40	8,923.16	8,860.73	0.20	−3,136.67
2d	882	2	2	1	−611,892.21	6,484.58	6,607.89	10,508.40	10,403.68	0.19	−3,242.29
2c	588	2	0	1	−680,931.04	7,353.56	7,928.15	9,735.63	9,585.98	0.09	−3,676.78
2b	735	2	2	0	−603,274.79	6,385.43	6,475.01	9,690.65	9,590.39	0.20	−3,192.71
2a	441	2	0	0	−663,487.89	7,118.36	7,727.43	8,829.62	8,740.71	0.12	−3,559.18
1d	735	1	2	1	−615,153.62	6,495.03	6,860.45	9,809.44	9,685.96	0.18	−3,247.51
1c	441	1	0	1	−683,013.53	7,175.04	7,284.49	9,024.87	8,928.94	0.09	−3,587.52
1b	588	1	2	0	−604,367.54	6,397.33	6,540.73	8,969.99	8,889.37	0.20	−3,198.66
1a	294	1	0	0	−656,065.24	6,892.42	7,065.18	8,023.81	7,928.68	0.13	−3,446.21

Model comparison table for acquisition CS- trials.

Model Comparison - Acquisition CS-											
Model #	Total Params	LR Params	Start Params	Jump Params	Log Likelihood	LOOIC	WAIC	BIC	Integrated BIC	Pseudo R^2	LOO
7d	1323	5	2	1	**−561,054.04**	**5,986.78**	6,355.74	12,194.76	12,106.35	**0.50**	**−2,993.39**
7c	1029	5	0	1	−564,918.40	6,047.85	6,404.84	10,770.24	10,673.09	0.50	−3,023.93
7b	1176	5	2	0	−566,482.46	6,020.87	**6,042.95**	11,517.46	11,422.00	0.50	−3,010.43
7a	882	5	0	0	−587,003.48	6,288.77	6,425.60	10,259.51	10,159.69	0.48	−3,144.38
6d	1029	3	2	1	−571,827.91	6,084.99	6,107.97	10,839.34	10,734.35	0.49	−3,042.49
6c	735	3	0	1	−586,998.74	6,223.17	6,244.48	9,527.89	9,446.54	0.48	−3,111.59
6b	882	3	2	0	−575,957.30	6,116.96	6,147.58	10,149.05	10,059.62	0.49	−3,058.48
6a	588	3	0	0	−606,293.41	6,404.06	6,398.90	**8,989.25**	8,897.08	0.46	−3,202.03
5d	882	2	2	1	−586,569.40	6,304.53	6,372.89	10,255.17	10,168.93	0.48	−3,152.26
5c	588	2	0	1	−651,316.45	7,106.33	7,218.75	9,439.48	9,326.94	0.42	−3,553.16
5b	735	2	2	0	−620,604.66	6,709.90	6,856.10	9,863.95	9,767.27	0.45	−3,354.95
5a	441	2	0	0	−681,618.34	7,519.28	7,673.84	9,010.92	**8,896.20**	0.40	−3,759.64
4d	1176	4	2	1	−587,035.94	6,235.64	6,344.28	11,723.00	11,641.71	0.48	−3,117.82
4c	882	4	0	1	−608,619.13	6,458.75	6,914.98	10,475.67	10,391.47	0.46	−3,229.37
4b	1029	4	2	0	−596,040.08	6,389.94	6,709.36	11,081.46	10,941.48	0.47	−3,194.97
4a	735	4	0	0	−629,977.12	6,730.48	6,958.09	9,957.67	9,858.13	0.44	−3,365.24
3d	1029	3	2	1	−585,403.13	6,250.50	6,511.22	10,975.09	10,870.09	0.48	−3,125.25
3c	735	3	0	1	−613,370.96	6,501.60	6,954.88	9,791.61	9,686.96	0.46	−3,250.80
3b	882	3	2	0	−595,419.39	6,352.13	6,501.19	10,343.67	10,229.35	0.47	−3,176.07
3a	588	3	0	0	−638,054.13	6,786.71	7,269.36	9,306.86	9,194.65	0.44	−3,393.36
2d	882	2	2	1	−611,059.66	6,716.21	7,671.59	10,500.08	10,357.60	0.46	−3,358.10
2c	588	2	0	1	−709,528.40	7,734.06	8,256.62	10,021.60	9,823.61	0.37	−3,867.03
2b	735	2	2	0	−619,579.75	6,764.78	7,460.26	9,853.70	9,729.19	0.45	−3,382.39
2a	441	2	0	0	−730,913.76	7,890.96	8,477.12	9,503.88	9,324.79	0.35	−3,945.48
1d	735	1	2	1	−621,731.78	6,699.83	7,646.24	9,875.22	9,729.41	0.45	−3,349.92
1c	441	1	0	1	−739,734.74	7,885.31	8,070.68	9,592.09	9,490.18	0.35	−3,942.65
1b	588	1	2	0	−658,869.35	7,174.52	7,881.05	9,515.01	9,372.40	0.42	−3,587.26
1a	294	1	0	0	−778,440.48	8,192.10	8,311.51	9,247.56	9,145.19	0.31	−4,096.05

Model comparison table for Extinction CS+ trials.

Model Comparison - Extinction CS+											
Model #	Total Params	LR Params	Start Params	Jump Params	Log Likelihood	LOOIC	WAIC	BIC	Integrated BIC	Pseudo R^2	LOO
7d	1323	5	2	1	−410,609.83	4,401.98	4,384.13	10,690.32	10,635.61	0.45	−2,200.99
7c	1029	5	0	1	−444,713.43	4,664.75	4,645.72	9,568.19	9,505.08	0.40	−2,332.38
7b	1176	5	2	0	−468,943.40	5,127.20	5,183.14	10,542.07	10,444.60	0.37	−2,563.60
7a	882	5	0	0	−485,657.28	5,253.49	5,414.09	9,246.05	9,130.40	0.35	−2,626.75
6d	1029	3	2	1	−429,917.83	4,564.72	4,550.14	9,420.24	9,376.15	0.42	−2,282.36
6c	735	3	0	1	−484,469.92	5,124.54	5,138.01	8,502.60	8,402.04	0.35	−2,562.27
6b	882	3	2	0	−463,688.56	4,953.12	4,953.13	9,026.36	8,952.63	0.38	−2,476.56
6a	588	3	0	0	−482,311.40	5,095.89	5,144.98	7,749.43	7,695.36	0.35	−2,547.95
5d	882	2	2	1	−453,091.41	4,782.36	4,799.98	8,920.39	8,844.96	0.39	−2,391.18
5c	588	2	0	1	−552,545.58	5,855.29	5,945.36	8,451.78	8,367.76	0.26	−2,927.65
5b	735	2	2	0	−479,198.92	5,215.70	5,507.80	8,449.89	8,351.42	0.35	−2,607.85
5a	441	2	0	0	−548,765.58	5,800.72	5,978.69	7,682.40	7,590.09	0.26	−2,900.36
4d	1176	4	2	1	**−410,015.94**	**4,387.78**	**4,367.89**	9,952.80	9,883.54	**0.45**	**−2,193.89**
4c	882	4	0	1	−442,611.43	4,655.78	4,681.39	8,815.59	8,768.26	0.40	−2,327.89
4b	1029	4	2	0	−465,945.95	5,071.58	5,096.91	9,780.52	9,672.16	0.37	−2,535.79
4a	735	4	0	0	−482,973.03	5,136.48	5,279.58	8,487.63	8,409.66	0.35	−2,568.24
3d	1029	3	2	1	−428,705.88	4,537.63	4,520.85	9,408.12	9,340.31	0.42	−2,268.82
3c	735	3	0	1	−482,636.75	5,091.88	5,065.45	8,484.27	8,408.28	0.35	−2,545.94
3b	882	3	2	0	−462,759.22	4,938.47	4,915.63	9,017.07	8,943.31	0.38	−2,469.23
3a	588	3	0	0	−479,220.86	5,058.60	5,162.90	7,718.53	7,616.24	0.35	−2,529.30
2d	882	2	2	1	−429,350.92	4,555.94	4,542.18	8,682.99	8,617.02	0.42	−2,277.97
2c	588	2	0	1	−478,072.62	5,033.45	5,119.57	7,707.05	7,616.62	0.36	−2,516.72
2b	735	2	2	0	−460,628.84	4,932.94	5,013.88	8,264.19	8,184.11	0.38	−2,466.47
2a	441	2	0	0	−483,350.09	5,051.22	5,060.19	7,028.24	6,982.01	0.35	−2,525.61
1d	735	1	2	1	−454,399.66	4,814.31	4,805.55	8,201.90	8,129.38	0.39	−2,407.16
1c	441	1	0	1	−521,158.02	5,499.96	5,566.19	7,406.32	7,333.49	0.30	−2,749.98
1b	588	1	2	0	−480,338.14	5,148.93	5,243.14	7,729.70	7,642.46	0.35	−2,574.46
1a	294	1	0	0	−519,416.50	5,436.57	5,485.50	**6,657.32**	**6,585.97**	0.30	−2,718.28

Model comparison table for Extinction CS- trials.

Model Comparison - Extinction CS-											
Model #	Total Params	LR Params	Start Params	Jump Params	Log Likelihood	LOOIC	WAIC	BIC	Integrated BIC	Pseudo R^2	LOO
7d	1323	5	2	1	**−374,345.05**	**4,040.92**	**4,034.26**	10,327.67	10,249.40	**0.67**	**−2,020.46**
7c	1029	5	0	1	−377,098.95	4,058.65	4,051.38	8,892.05	8,824.84	0.67	−2,029.32
7b	1176	5	2	0	−469,099.96	5,047.68	5,099.97	10,543.64	10,468.75	0.59	−2,523.84
7a	882	5	0	0	−514,633.36	5,580.10	5,973.38	9,535.81	9,414.25	0.55	−2,790.05
6d	1029	3	2	1	−389,199.30	4,195.60	4,192.51	9,013.05	8,947.74	0.66	−2,097.80
6c	735	3	0	1	−408,346.92	4,484.56	4,592.46	7,741.37	7,664.22	0.64	−2,242.28
6b	882	3	2	0	−486,940.93	5,219.46	5,237.86	9,258.89	9,198.23	0.57	−2,609.73
6a	588	3	0	0	−531,782.85	5,658.98	5,739.44	8,244.15	8,154.91	0.53	−2,829.49
5d	882	2	2	1	−400,319.31	4,247.61	4,233.29	8,392.67	8,310.08	0.65	−2,123.81
5c	588	2	0	1	−421,386.31	4,497.30	4,491.94	7,140.18	7,063.29	0.63	−2,248.65
5b	735	2	2	0	−549,361.77	5,943.68	6,602.62	9,151.52	9,039.54	0.51	−2,971.84
5a	441	2	0	0	−562,195.09	5,990.19	6,294.86	7,816.69	7,709.15	0.50	−2,995.10
4d	1176	4	2	1	−380,069.23	4,103.29	4,093.46	9,653.33	9,577.71	0.66	−2,051.64
4c	882	4	0	1	−387,831.43	4,197.56	4,193.01	8,267.79	8,186.81	0.66	−2,098.78
4b	1029	4	2	0	−475,447.97	5,114.62	5,137.98	9,875.54	9,798.06	0.58	−2,557.31
4a	735	4	0	0	−517,097.97	5,572.92	5,953.88	8,828.88	8,725.03	0.54	−2,786.46
3d	1029	3	2	1	−391,222.30	4,216.82	4,215.28	9,033.28	8,973.01	0.65	−2,108.41
3c	735	3	0	1	−413,238.34	4,546.70	4,580.23	7,790.28	7,719.71	0.63	−2,273.35
3b	882	3	2	0	−489,309.76	5,185.44	5,177.48	9,282.58	9,217.05	0.57	−2,592.72
3a	588	3	0	0	−536,043.30	5,666.65	5,942.52	8,286.75	8,158.03	0.53	−2,833.33
2d	882	2	2	1	−397,457.16	4,337.79	4,390.23	8,364.05	8,285.67	0.65	−2,168.90
2c	588	2	0	1	−444,531.56	4,896.61	5,086.35	7,371.64	7,262.51	0.61	−2,448.31
2b	735	2	2	0	−496,533.01	5,307.95	5,472.95	8,623.23	8,531.50	0.56	−2,653.98
2a	441	2	0	0	−548,069.89	5,799.88	5,926.49	7,675.44	7,589.95	0.52	−2,899.94
1d	735	1	2	1	−408,335.51	4,356.33	4,354.50	7,741.25	7,666.90	0.64	−2,178.17
1c	441	1	0	1	−455,309.19	4,960.26	5,049.96	**6,747.83**	**6,658.17**	0.60	−2,480.13
1b	588	1	2	0	−553,117.71	5,894.09	6,187.96	8,457.50	8,360.38	0.51	−2,947.05
1a	294	1	0	0	−579,532.04	6,084.70	6,302.42	7,258.48	7,168.26	0.49	−3,042.35

Associations with corrections

Partial Spearman correlation, correcting for CS pattern. (Was one of the four pseudorandom CS patterns in some way influential on the association?)

	Medium GAD-7		Medium PHQ-8		Strict GAD-7		Strict PHQ-8	
Parameter	Regular	CS Pattern	Regular	CS Pattern	Regular	CS Pattern	Regular	CS Pattern
Acquisition CS+ Learning Rate (US+)	-0.07 [-0.23, 0.10]	-0.09 [-0.25, 0.08]	-0.03 [-0.20, 0.13]	-0.04 [-0.21, 0.12]	-0.15 [-0.36, 0.08]	-0.16 [-0.36, 0.06]	-0.01 [-0.22, 0.20]	-0.01 [-0.23, 0.20]
Acquisition CS+ Learning Rate (US-)	-0.10 [-0.27, 0.07]	-0.11 [-0.27, 0.07]	-0.06 [-0.23, 0.12]	-0.06 [-0.22, 0.10]	**-0.30 [-0.50, -0.07]**	**-0.30 [-0.50, -0.09]**	-0.20 [-0.40, 0.00]	-0.20 [-0.41, 0.02]
Acquisition CS- Learning Rate	**-0.22 [-0.36, -0.06]**	**-0.22 [-0.37, -0.06]**	-0.14 [-0.29, 0.02]	-0.14 [-0.28, 0.02]	**-0.32 [-0.49, -0.12]**	**-0.32 [-0.50, -0.11]**	-0.14 [-0.33, 0.05]	-0.14 [-0.33, 0.06]
Extinction CS+ Learning Rate	**-0.21 [-0.37, -0.06]**	**-0.23 [-0.38, -0.06]**	**-0.23 [-0.38, -0.06]**	**-0.23 [-0.39, -0.06]**	**-0.33 [-0.52, -0.11]**	**-0.34 [-0.53, -0.13]**	**-0.26 [-0.46, -0.06]**	**-0.26 [-0.45, -0.05]**
Extinction CS- Learning Rate	-0.07 [-0.22, 0.09]	-0.07 [-0.23, 0.09]	-0.10 [-0.27, 0.07]	-0.10 [-0.26, 0.07]	-0.15 [-0.35, 0.08]	-0.15 [-0.36, 0.07]	-0.13 [-0.34, 0.10]	-0.13 [-0.34, 0.10]
Lapse Rate	-0.06 [-0.21, 0.11]	-0.06 [-0.21, 0.10]	-0.16 [-0.31, 0.01]	-0.16 [-0.30, 0.02]	-0.13 [-0.33, 0.09]	-0.13 [-0.33, 0.09]	**-0.22 [-0.42, -0.00]**	**-0.22 [-0.42, -0.00]**

Bold ~ p < 0.05

Regular = no controls; CS Pattern = controlling for CS pattern

GAD-7 - Generalized Anxiety Disorder seven item scale; PHQ-8 - Patient Health Questionnaire eight item scale

CS pattern distribution: 1 = 31, 2 = 40, 3 = 37, 4 = 37

Medium dataset: CS pattern correlations GAD-7 ρ=-0.08, PHQ-8 ρ=-0.02

Strict dataset: CS pattern correlations GAD-7 ρ=0.00, PHQ-8 ρ=-0.03

Partial Spearman correlation, correcting for age, sex, and both combined.

	Medium GAD-7				Medium PHQ-8				Strict GAD-7				Strict PHQ-8			
Parameter	Regular	Age	Sex	Both	Regular	Age	Sex	Both	Regular	Age	Sex	Both	Regular	Age	Sex	Both
Acquisition CS+ Learning Rate (US+)	-0.07 [-0.24, 0.10]	-0.07 [-0.23, 0.11]	-0.07 [-0.24, 0.08]	-0.07 [-0.24, 0.09]	-0.03 [-0.19, 0.13]	-0.04 [-0.21, 0.12]	-0.04 [-0.21, 0.13]	-0.04 [-0.21, 0.12]	-0.15 [-0.36, 0.05]	-0.15 [-0.37, 0.07]	-0.16 [-0.37, 0.06]	-0.16 [-0.37, 0.07]	-0.01 [-0.22, 0.21]	-0.01 [-0.23, 0.21]	-0.01 [-0.22, 0.20]	-0.01 [-0.24, 0.22]
Acquisition CS+ Learning Rate (US-)	-0.10 [-0.26, 0.07]	-0.08 [-0.26, 0.08]	-0.08 [-0.25, 0.09]	-0.07 [-0.24, 0.11]	-0.06 [-0.22, 0.12]	-0.03 [-0.19, 0.15]	-0.05 [-0.22, 0.12]	-0.02 [-0.20, 0.15]	**-0.30 [-0.50, -0.07]**	**-0.28 [-0.50, -0.05]**	**-0.29 [-0.47, -0.06]**	**-0.27 [-0.47, -0.06]**	-0.20 [-0.40, 0.00]	-0.17 [-0.37, 0.05]	-0.18 [-0.41, 0.04]	-0.16 [-0.37, 0.07]
Acquisition CS- Learning Rate	**-0.22 [-0.37, -0.05]**	**-0.22 [-0.37, -0.05]**	**-0.22 [-0.37, -0.05]**	**-0.22 [-0.36, -0.05]**	-0.14 [-0.29, 0.03]	-0.13 [-0.30, 0.03]	-0.14 [-0.30, 0.03]	-0.13 [-0.29, 0.03]	**-0.32 [-0.49, -0.12]**	**-0.31 [-0.49, -0.10]**	**-0.32 [-0.49, -0.12]**	**-0.31 [-0.50, -0.10]**	-0.14 [-0.33, 0.05]	-0.10 [-0.30, 0.11]	-0.15 [-0.34, 0.07]	-0.11 [-0.32, 0.12]
Extinction CS+ Learning Rate	**-0.21 [-0.36, -0.05]**	**-0.21 [-0.36, -0.04]**	**-0.21 [-0.37, -0.05]**	**-0.21 [-0.37, -0.04]**	**-0.23 [-0.38, -0.07]**	**-0.23 [-0.39, -0.05]**	**-0.22 [-0.38, -0.05]**	**-0.23 [-0.38, -0.06]**	**-0.33 [-0.52, -0.14]**	**-0.33 [-0.52, -0.11]**	**-0.33 [-0.52, -0.11]**	**-0.32 [-0.52, -0.12]**	**-0.26 [-0.44, -0.05]**	**-0.25 [-0.45, -0.03]**	**-0.25 [-0.43, -0.05]**	**-0.24 [-0.43, -0.04]**
Extinction CS- Learning Rate	-0.07 [-0.23, 0.10]	-0.09 [-0.25, 0.06]	-0.06 [-0.22, 0.11]	-0.08 [-0.25, 0.08]	-0.10 [-0.25, 0.07]	-0.12 [-0.28, 0.06]	-0.09 [-0.25, 0.08]	-0.12 [-0.28, 0.04]	-0.15 [-0.35, 0.07]	-0.19 [-0.39, 0.05]	-0.14 [-0.34, 0.10]	-0.17 [-0.38, 0.06]	-0.13 [-0.36, 0.09]	-0.17 [-0.38, 0.06]	-0.12 [-0.34, 0.10]	-0.15 [-0.36, 0.07]
Lapse Rate	-0.06 [-0.21, 0.12]	-0.06 [-0.23, 0.11]	-0.05 [-0.23, 0.10]	-0.06 [-0.23, 0.11]	-0.16 [-0.32, 0.00]	-0.15 [-0.32, 0.02]	-0.15 [-0.30, 0.01]	-0.15 [-0.29, 0.02]	-0.13 [-0.33, 0.09]	-0.14 [-0.33, 0.10]	-0.13 [-0.32, 0.10]	-0.13 [-0.35, 0.09]	**-0.22 [-0.41, -0.01]**	-0.21 [-0.42, 0.01]	**-0.21 [-0.41, 0.01]**	-0.21 [-0.40, -0.00]

Bold ~ p < 0.05

Regular = no controls; Age = controlling for age; Sex = controlling for sex; Both = controlling for age and sex

GAD-7 - Generalized Anxiety Disorder seven item scale; PHQ-8 - Patient Health Questionnaire eight item scale

Medium dataset (n=145): Age correlations GAD-7 ρ=-0.01, PHQ-8 ρ=-0.09; Sex correlations GAD-7 ρ=0.11, PHQ-8 ρ=0.06

Strict dataset (n=88): Age correlations GAD-7 ρ=-0.06, PHQ-8 ρ=-0.21; Sex correlations GAD-7 ρ=0.09, PHQ-8 ρ=0.11

Partial Spearman correlation, correcting for US unpleasantness rating in medium excluded dataset (n=145)

	GAD-7		PHQ-8	
Parameter	Regular ρ [95% CI]	Partial ρ [95% CI]	Regular ρ [95% CI]	Partial ρ [95% CI]
Acquisition CS+ Learning Rate (US+)	-0.07 [-0.24, 0.11]	-0.09 [-0.25, 0.08]	-0.03 [-0.20, 0.13]	-0.06 [-0.23, 0.12]
Acquisition CS+ Learning Rate (US-)	-0.10 [-0.25, 0.07]	-0.09 [-0.23, 0.08]	-0.06 [-0.22, 0.12]	-0.04 [-0.21, 0.13]
Acquisition CS- Learning Rate	**-0.22 [-0.37, -0.05]**	**-0.23 [-0.37, -0.06]**	-0.14 [-0.30, 0.02]	-0.15 [-0.30, 0.01]
Extinction CS+ Learning Rate	**-0.21 [-0.37, -0.05]**	**-0.21 [-0.36, -0.05]**	**-0.23 [-0.37, -0.06]**	**-0.22 [-0.37, -0.06]**
Extinction CS- Learning Rate	-0.07 [-0.23, 0.09]	-0.06 [-0.22, 0.10]	-0.10 [-0.26, 0.07]	-0.08 [-0.24, 0.08]
Lapse	-0.06 [-0.21, 0.10]	-0.03 [-0.20, 0.14]	-0.16 [-0.32, 0.00]	-0.13 [-0.28, 0.05]

Bold ~ p < 0.05

Regular = standard Spearman correlation; Partial = controlling for scream unpleasantness

GAD-7 - Generalized Anxiety Disorder seven item scale; PHQ-8 - Patient Health Questionnaire eight item scale

Scream unpleasantness correlations: GAD-7 ρ = 0.11 (p = 0.191), PHQ-8 ρ = 0.14 (p = 0.084)

Partial Spearman correlation, correcting for US unpleasantness rating in strict excluded dataset (n=88). This did remove the association between the lapse parameter and PHQ-8.

	GAD-7		PHQ-8	
Parameter	Regular ρ [95% CI]	Partial ρ [95% CI]	Regular ρ [95% CI]	Partial ρ [95% CI]
Acquisition CS+ Learning Rate (US+)	-0.15 [-0.36, 0.06]	-0.17 [-0.37, 0.06]	-0.01 [-0.22, 0.20]	-0.02 [-0.24, 0.20]
Acquisition CS+ Learning Rate (US-)	**-0.30 [-0.49, -0.08]**	**-0.29 [-0.49, -0.06]**	-0.20 [-0.38, 0.02]	-0.19 [-0.39, 0.02]
Acquisition CS- Learning Rate	**-0.32 [-0.48, -0.13]**	**-0.32 [-0.49, -0.14]**	-0.14 [-0.34, 0.07]	-0.14 [-0.33, 0.05]
Extinction CS+ Learning Rate	**-0.33 [-0.51, -0.14]**	**-0.33 [-0.52, -0.12]**	**-0.26 [-0.43, -0.03]**	**-0.25 [-0.43, -0.04]**
Extinction CS- Learning Rate	-0.15 [-0.34, 0.08]	-0.14 [-0.33, 0.08]	-0.13 [-0.35, 0.10]	-0.12 [-0.34, 0.09]
Lapse	-0.13 [-0.36, 0.07]	-0.12 [-0.32, 0.10]	**-0.22 [-0.41, 0.00]**	-0.20 [-0.38, 0.02]

Bold ~ p < 0.05

Regular = standard Spearman correlation; Partial = controlling for scream unpleasantness

GAD-7 - Generalized Anxiety Disorder seven item scale; PHQ-8 - Patient Health Questionnaire eight item scale

Scream unpleasantness correlations: GAD-7 ρ = 0.09 (p = 0.431), PHQ-8 ρ = 0.11 (p = 0.289)

Residuals analysis

	Medium Criteria (n=145)					Strict Criteria (n=88)				
Parameter	GAD-7	PHQ-8	GAD-7 residual	PHQ-8 residual	Shared variance	GAD-7	PHQ-8	GAD-7 residual	PHQ-8 residual	Shared variance
Acquisition CS+ Learning Rate (US+)	-0.07 [-0.23, 0.10]	-0.03 [-0.20, 0.13]	-0.03 [-0.21, 0.15]	0.02 [-0.13, 0.18]	-0.05 [-0.22, 0.11]	-0.15 [-0.36, 0.05]	-0.01 [-0.20, 0.21]	-0.16 [-0.39, 0.08]	0.13 [-0.10, 0.34]	-0.07 [-0.27, 0.16]
Acquisition CS+ Learning Rate (US-)	-0.10 [-0.26, 0.06]	-0.06 [-0.22, 0.10]	-0.06 [-0.24, 0.12]	-0.01 [-0.17, 0.16]	-0.08 [-0.23, 0.09]	**-0.30 [-0.50, -0.08]**	-0.20 [-0.40, 0.03]	-0.21 [-0.41, 0.02]	0.03 [-0.19, 0.25]	**-0.26 [-0.45, -0.05]**
Acquisition CS- Learning Rate	**-0.22 [-0.37, -0.06]**	-0.14 [-0.29, 0.02]	-0.14 [-0.29, 0.04]	0.01 [-0.16, 0.19]	**-0.18 [-0.34, -0.02]**	**-0.32 [-0.50, -0.11]**	-0.14 [-0.33, 0.06]	**-0.28 [-0.46, -0.07]**	0.16 [-0.04, 0.36]	**-0.22 [-0.41, -0.02]**
Extinction CS+ Learning Rate	**-0.21 [-0.39, -0.05]**	**-0.23 [-0.38, -0.06]**	-0.06 [-0.22, 0.10]	-0.09 [-0.26, 0.08]	**-0.24 [-0.39, -0.09]**	**-0.33 [-0.51, -0.13]**	**-0.26 [-0.44, -0.04]**	-0.17 [-0.38, 0.05]	-0.03 [-0.25, 0.19]	**-0.31 [-0.48, -0.12]**
Extinction CS- Learning Rate	-0.07 [-0.21, 0.09]	-0.10 [-0.26, 0.07]	0.06 [-0.10, 0.21]	-0.09 [-0.24, 0.07]	-0.10 [-0.25, 0.07]	-0.15 [-0.36, 0.07]	-0.13 [-0.35, 0.09]	0.05 [-0.16, 0.25]	-0.08 [-0.30, 0.13]	-0.16 [-0.35, 0.04]

Bold ~ p < 0.05

Values show Spearman correlation ρ [95% CI]. GAD-7 and PHQ-8 correlations ~ Medium ρ = 0.71, Strict ρ = 0.74

GAD-7 - Generalized Anxiety Disorder seven item scale; PHQ-8 - Patient Health Questionnaire eight item scale

Median split

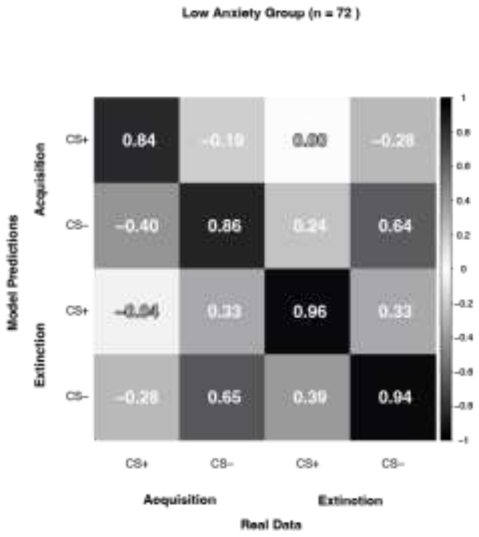

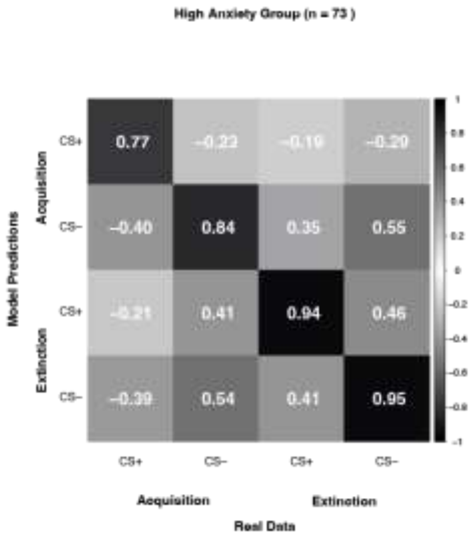

Supplement: Supplementary Materials. — Supplementary Methods & Supplementary Results. [file cpsy-10-1-138-s1.zip › cpsy-138_kerr/68b6a9e07c5d6.pdf]
